# ITLN1, orchestrated by the IFNγ-IRF1 axis, suppresses hepatocellular carcinoma proliferation via ERK1/2 activation

**DOI:** 10.1016/j.tranon.2025.102600

**Published:** 2025-11-11

**Authors:** Tong Yuan, Junjie Liu, Ronghua Zhu, Jiang Li, Zhiyong Huang, Huifang Liang, Haisu Tao, Erlei Zhang

**Affiliations:** a Hepatic Surgery Center, Tongji Hospital, Tongji Medical College, Huazhong University of Science and Technology, Wuhan, 430000, China; b Hepatobiliary Surgery, The First Affiliated Hospital, College of Medicine, Shihezi University, Shihezi, 832000, China; c Department of Hepatobiliary Surgery, Zhujiang Hospital, Southern Medical University, Guangzhou, 510280, China

**Keywords:** Hepatocellular carcinoma, ITLN1, ERK1/2, IRF1, IFNγ

## Abstract

**Background:**

Intelectin 1 (ITLN1) is a recently discovered secretory adipokine with pivotal functions in the innate immune system, inflammation, and the facilitation of glucose uptake. Nonetheless, its exact functions in hepatocellular carcinoma (HCC) remain not fully elucidated.

**Methods:**

In this study, ITLN1 was identified as a clinically significant secretory adipokine linked to HCC, validated through qRT-PCR, western blot, immunohistochemistry, and TCGA data. Its role in HCC was explored using CCK-8, clone formation, EdU, migration, and cell cycle assays, alongside xenograft tumor experiments. RNA sequencing, luciferase reporter assays, and ChIP assays confirmed ITLN1′s molecular mechanisms in inhibiting HCC proliferation.

**Results:**

Our study revealed that ITLN1 expression was significantly downregulated in HCC tissues compared to adjacent non-tumor tissues, and its reduced expression was associated with poor overall survival. Functionally, ITLN1 attenuated HCC proliferation in a cell cycle arrest manner via activation of ERK1/2 signaling. We also identified transcription factor interferon regulatory factor 1 (IRF1) as a regulator of ITLN1 through bioinformatics analysis and affirmed the binding site on the ITLN1 promoter. Furthermore, interferon-gamma (IFNγ), a classic upstream cytokine of IRF1, could promote ITLN1 expression through IRF1. Subsequently, the IFNγ-IRF1-ITLN1 axis was identified and found to inhibit HCC cell proliferation and cell cycle progression.

**Conclusions:**

In summary, our study found that ITLN1, regulated by IFNγ-IRF1 axis, suppresses HCC proliferation by constitutively activating ERK1/2 signaling and holds promise as a prospective prognostic indicator and a plausible therapeutic target for HCC.

## Highlights

• ITLN1 acts as a tumor suppressor in hepatocellular carcinoma (HCC).
• Decreased expression of ITLN1 was indicative of poor overall survival in HCC.
• ITLN1 attenuates HCC proliferation and induces cell cycle arrest.
• ITLN1 protects against HCC via activation of ERK1/2 signaling.
• IFNγ-IRF1-ITLN1 axis inhibits HCC cells proliferation and cell cycle progression.

## Introduction

The burden of hepatocellular carcinoma (HCC) has been steadily increasing over the past few decades, and metabolic risk factors are gradually becoming major contributing factors to HCC [1]. During hepatocarcinogenesis associated with obesity, dysregulation of adipokine secretion occurs, activating multiple signaling pathways. However, adipokines are diverse and can both promote and inhibit the development of HCC in different settings [[2], [3], [4]]. Hence, further investigation is essential to comprehend the distinct roles of different adipokines in the onset and advancement of HCC.

Intelectin 1 (ITLN1), an adipokine and lectin recently identified, exhibits predominant expression in adipose tissue and gastrointestinal tract [5]. The expression of ITLN1 shows significant variability among patients with different types of cancer. Circulating ITLN1 concentrations are elevated in gastrointestinal and prostate cancers but lower in gynecologic, breast, bladder, and kidney cancers compared to healthy volunteers [5]. ITLN1 was proven to exerts a protective influence in neuroblastoma and gastrointestinal tumors. What’s more, it acts as a modulator influencing the invasive capacity and metabolic processes of ovarian cancer cells [[6], [7], [8], [9]]. Nevertheless, the specific role of ITLN1 in HCC remains uncertain.

Our research revealed a downregulation of ITLN1 expression in HCC tissues compared to adjacent non-tumor tissues, and this downregulation corrected with an unfavorable prognosis for patients with HCC [10]. Functionally, ITLN1 functioned as a tumor suppressor through the activation of the ERK1/2 signaling pathway, leading to the inhibition of HCC growth. Furthermore, IFNγ was identified to upregulate ITLN1 expression through the classical downstream molecule IRF1 in a transcriptional regulatory manner. The IFNγ-IRF1-ITLN1 axis was identified as a regulatory pathway influencing HCC cell proliferation.

## Materials and methods

### Clinical specimens

The Tongji cohort comprises 95 paired specimens of tumor and adjacent non-tumor tissues obtained from HCC patients who underwent hepatectomy at Tongji Hospital between 2015 and 2016. Detailed information on all cancer patients is provided in Table S1. Written informed consent was acquired from all patients, and ethical approval for each procedure was granted by the Ethical Review Committee of Tongji Hospital.

### Reagents and antibodies

PD98059, an inhibitor of Mitogen-activated protein kinase (MEK) 1/2 was purchased from MedChem Express (# HY-12028, Monmouth Junction, NJ, USA), and recombinant Human IFNγ was acquired from PeproTech (#300-02, Thermo Fisher Scientific, MA, USA). The antibodies utilized are detailed in Table S2.

### Cell lines and culture conditions

We purchased the 293T, HepG2, and multiple HCC cell lines (HLE, HLF, Huh7, HCC-LM3, PLC/PRF/5, MHCC-97H, SK-Hep1, and SNU449) from the China Center for Type Culture Collection (CCTCC). All cell lines were maintained in Dulbecco's Modified Eagle medium (DMEM) (Thermo Fisher Hyclone, Shanghai, China) containing 10 % fetal bovine serum (FBS) (Thermo Fisher Hyclone, Shanghai, China) at 37 °C in a 5 % CO2 and 95 % air atmosphere.

### Gene knockdown and siRNA transfection

To construct pLKO.1-scramble and pLKO.1-shITLN1 plasmids, a non-targeting sequence and the target oligonucleotide were cloned into the pLKO.1 plasmid (Addgene #10,878). Lentivirus packaging, cell infection, and stable clone screening were conducted as previously described [11]. And, siRNAs (RiboBio, Guangzhou, China) targeting ITLN1, IRF1, and their respective negative control were transfected when HCC cells reached 70 % confluence. The specific sequences for shRNA and siRNA used are outlined in Table S3.

### Gene overexpression and reintroduction

Human ITLN1 cDNA (NM_017625.3) and IRF1 cDNA (NM_001354924.1) were amplified and inserted into pLenti-CMV-Puro plasmids (Addgene #17,448) at *Bam*HI/SalI sites or pcDNA3.1 plasmids (Addgene #73,066) at HindIII/*Bam*HI sites, resulting in pLenti-ITLN1, pcDNA3.1-ITLN1, and pcDNA3.1-IRF1 plasmids. Lentivirus packaging and stable ITLN1 overexpression were achieved using pLenti-ITLN1, as described [11]. Transient overexpression of ITLN1 and IRF1 was carried out by transfecting pcDNA3.1-ITLN1 into IRF1-si SK-Hep1 cells and pcDNA3.1-IRF1 into wild-type HCC cells.

### Cell counting kit-8 (CCK-8) assay and colony formation assay

HCC cells (800 cells/well) were seeded in 96-well and 6-well plates and cultured in complete medium. Cell viability was measured using the CCK-8 assay at designated time points, with five replicates per group. After 2 weeks, colonies were fixed, stained, and counted using Image J 1.8.0 (NIH, USA), considering only those with a diameter >100 µm.

### 5-Ethynyl-2′-deoxyuridine (EdU) incorporation assay

The EdU incorporation assay was performed using the Cell-Light™ EdU Apollo567 In Vitro Flow Cytometry Kit (RiboBio, Guangzhou, China). HCC cells (3000 cells/well) were seeded in 96-well plates overnight, treated with EdU, fixed with 4 % paraformaldehyde, and permeabilized with 0.5 % Triton X-100. After incubation with 1 × Apollo reaction cocktail for 30 min, nuclei were stained with Hoechst and visualized under a fluorescence microscope.

### Wound healing assay and cell migration assay

For wound healing assay, HCC cells were cultured until reaching 90 % confluence in DMEM with 10 % FBS. A controlled scratch was made and the migration was observed at 0 and 24 h post-scratch. For cell migration assay, transwell chambers (Corning, NY, USA) was utilized. HCC cells (3–6 × 10^4^) in 100 μL FBS-free DMEM were added to upper chambers, the lower chambers contained DMEM with 10 % FBS as the chemoattractant. After a 24-hour incubation period, migrated cells were fixed, stained with 0.1 % crystal violet, and quantified from five random fields using light microscopy. Image J 1.8.0 was used to analyse the results.

### Cell cycle analysis

HCC cells were digested, rinsed, and subsequently fixed with cold 70 % ethanol. Following washing and centrifugation, the cells were treated with propidium iodide (PI) containing RNase A for 30 min. The cell cycle distribution was detected with Flow cytometry (BD Biosciences, San Diego, CA, USA). Triplicate experiments were conducted to in each group.

### Luciferase reporter assay

The ITLN1 promoter including 1 kilobase upstream and 0.1 kilobase downstream sequence of the transcription start site (TSS) was amplified by AuGCTBiotechnology Co., Ltd (Beijing, China), cloned into the pGL4.17[luc2/Neo] Vector plasmid (Promega, Madison, WI, USA) at the KpnI/XhoI locus and named pGL4.17-ITLN1 promoter (WT) plasmid. Then pGL4.17-ITLN1 promoter (M1), pGL4.17-ITLN1 promoter (M2), pGL4.17-ITLN1 promoter (M3), and pGL4.17-ITLN1 promoter (M4) plasmids were individually constructed by introducing mutations into each of the possible IRF1 binding sites. HCC cells (1 × 10^5^ cells/well) were seeded in 24-well plates and co-transfected with three kinds of plasmids: (1) 0.2 μg expression construct pcDNA3.1-vector and pcDNA3.1-IRF1; (2) 0.48 μg reporter construct pGL4.17-vector, pGL4.17-ITLN1 promoter (WT), pGL4.17-ITLN1 promoter (M1), pGL4.17-ITLN1promoter (M2), pGL4.17-ITLN1 promoter (M3), pGL4.17-ITLN1 promoter (M4); and (3) 20 ng control reporter pRL-SV 40 (Promega, Madison, WI, USA). After co-transfection for 36 h, luciferase activities were measured using GloMax 20/20 Luminometer (Promega, Madison, WI, USA). The data are presented as the ratio of firefly luciferase values to Renilla luciferase values.

### Chromatin immunoprecipitation (ChIP) assay

The SimpleChIP Plus Sonication Chromatin IP kit (#56,383, Cell Signaling Technology, Beverly, MA, USA) was used to perform the ChIP assay. In brief, HCC cells were cross-linked, lysed and the chromatin was sonicated into 200–500 bp fragments. The fragmented chromatin was then subjected to incubation with antibodies specific to IgG, HA, or IRF1 at 4 °C overnight. Following the degradation of proteins in the precipitated complexes using proteinase K, the levels of immunoprecipitated DNA were quantified through qRT-PCR. Refer to Table S4 for ChIP primer sequences.

### Immunohistochemistry (IHC)

The protein expressions of ITLN1, IRF1, and IFNγ in human HCC tissues as well as p-ERK in mice tumor were evaluated in IHC assay. Briefly, the tissue slides underwent dewaxing, rehydration, antigen repair, and antigen blocking. Following the antigen blocking step, the slides were then incubated with primary antibodies and secondary antibody. Finally, the slides were dyed with the 3-diaminobenzidine tetra-hydrochloride (DAB) and hematoxylin. The concrete laboratory procedure and staining scores were described as previously reported [12]. Specifically, all 18 patients assessed for ITLN1 protein expression by Western blot also underwent ITLN1 IHC staining, and representative images from two typical patients were selected for presentation.

### Western blot

Total proteins of human cells or tissues were extracted using RIPA buffer. For culture supernatant proteins, concentration was achieved using a 10,000 MWCO spin column (Millipore, Billerica, MA, USA). The subsequent experimental steps and reagents used are referenced from previous study [12]. The details of the antibodies used are available in Table S2.

### qRT-PCR assay

In qRT-PCR assay, TRIzol Reagent (Invitrogen, Life Technologies, Carlsbad, CA, USA) was utilized to extract total RNA. And QuantScript RT Kit (TIANGEN, Beijing, China) was used to synthesize the cDNA. Subsequently, qRT-PCR was carried out, with each experiment conducted in triplicate. For data analysis, housekeeping gene β-actin was employed as an endogenous control. The detailed methodology and the list of reagents used in qRT-PCR can be found in previous study [12]. The primer details are outlined in Table S4.

### Xenograft tumor growth assay in nude mice

Male Balb/c athymic nude mice aged 4–6 weeks were procured from HFK Bioscience Co. Ltd. (Beijing, China). For the purpose of assessing subcutaneous tumor growth in the nude mice, a total of 1 × 10^6^ MHCC-97H-ITLN1 or MHCC-97H-vector cells were injected subcutaneously into the lateral abdomen (5 mice/group). Tumor volumes were meticulously observed and assessed every 5 days using the formula: Volume (mm^3^) = 0.5 × *L* (length, mm) × W^2^ (width, mm^2^). The mice were sacrificed 30 days after tumor transplantation and then tumors were excised, photographed and weighed. To monitor orthotopic tumor growth in vivo, 1 × 10^6^ MHCC-97H-ITLN1 or MHCC-97H-vector cells were injected into the liver of nude mice (5 mice /group). For the evaluation of tumor metastasis in vivo, mice (5 mice/group) were injected with a total of 1 × 10^6^ MHCC-97H-ITLN1 or MHCC-97H vector cells into the tail vein. The study utilized bioluminescence imaging to monitor the development of liver tumors and lung metastases. The mice were sacrificed 6 weeks post-transplantation. After fixation with 4 % paraformaldehyde, all tumors from the aforementioned models were then subjected to IHC analysis.

### RNA sequencing

Total RNA was extracted following the previously described method and sent to Bioyigene Biotechnology Co., Ltd. (Wuhan, China) to construct the library and perform high-throughput sequencing[12]. After ITLN1 knockdown in Huh7 cells, RNA sequencing was performed to compare transcriptomic profiles between ITLN1-scramble and ITLN1-knockdown groups. Differentially expressed genes were defined as those with |log2FC| ≥ 1 and false discovery rate (FDR) ≤ 0.05, and the screened genes were subsequently subjected to downstream analyses.

### Statistics analysis

For statistical analyses, we utilized GraphPad Prism 8.0 (GraphPad Software Inc., La Jolla, CA, USA), R (version 3.6.1) and SPSS (standard V.16.0; IBM Corporation, Armonk, NY, USA). Unless otherwise specified, all experiments included 3 replicates and the results were presented as means ± standard deviation (SD). The type of statistical analysis employed depended on the nature of the experiments. The appropriate statistical tests, including the analysis of variance (ANOVA), two-tailed student's *t*-test, or nonparametric test, were employed for comparisons as needed. The study compared patient characteristics and ITLN1 expression levels using the Chi-square (χ2) test. The relationships between ITLN1, IRF1, and IFNγ in HCC were evaluated using the Pearson’s correlation test. For survival analysis, we employed the Kaplan-Meier survival curve and the log-rank test. A *p* value < 0.05 was considered statistically significant, with * denoting *p* < 0.05, ** for *p* < 0.01, and *** for *p* < 0.001.

Kaplan–Meier survival analyses were performed using the “survival” and “sur*vminer*” packages in R. For TCGA cohort, overall survival (OS) was analyzed. Patients with ITLN1 TPM = 0 were excluded to ensure reliable stratification. The optimal cut-off value for ITLN1 expression (TPM = 0.167) was determined using the “sur*vminer*” package, and patients were stratified into high- and low-expression groups accordingly. For the Tongji cohort, ITLN1 mRNA expression was quantified by qRT-PCR and normalized to β-actin expression. Relative expression levels were calculated using the 2^-ΔCt method. Both overall survival (OS) and recurrence-free survival (RFS) were analyzed, with the optimal cut-off value again determined by the “sur*vminer*” package to stratify patients into high- and low-expression groups.

## Results

### Identification of ITLN1 as a clinically significant secretory adipokine associated with HCC

**Fig. 1:**
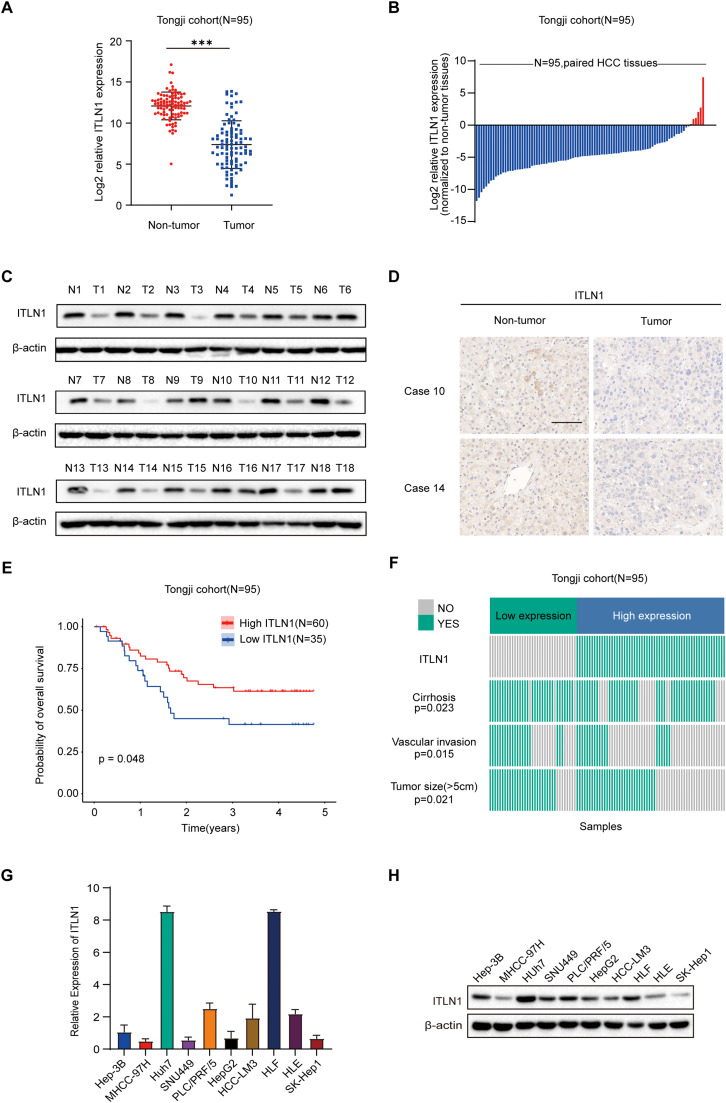
Identification of ITLN1 as a clinically significant secretory adipokine associated with HCC. (A) qRT-PCR analysis of ITLN1 expression of 95 paired HCC tissues in Tongji cohort. (B) Wilcoxon matched-pairs test compared ITLN1 mRNA levels between HCC tumor and adjacent non-tumor tissues. (C) Western blot analysis of ITLN1 protein level in 18 paired HCC tumor tissues (T) and corresponding adjacent non-tumor tissues (N). (D) Protein expression of ITLN1 in paired HCC samples from Tongji cohort was examined using immunohistochemical staining. Representative two samples stained for ITLN1 were shown. Scale bars, 100 μm. (E) Kaplan–Meier survival analysis evaluated ITLN1 impact on overall survival. (F) Chi-square analysis showed a negative correlation between ITLN1 expression and tumor size, as well as the risks of cirrhosis and vascular invasion (Tongji cohort). (G) The expression level of ITLN1 mRNA in HCC cell lines were investigated using qRT-PCR. (H) Western blot analysis of ITLN1 protein level in HCC cell lines. In (A, G, and H), data are from three independent repetitive experiments and shown as mean ± SD. *p < 0.05, **p < 0.01, ***p < 0.001.

RNA microarray analysis was performed on three paired HCC tumor and adjacent non-tumor samples to identify secretory proteins that correlate with the clinical features of HCC. A total of 109 differentially expressed secreted proteins were identified between tumor and adjacent non-tumor tissues, based on a fold change greater than 2 and *p* < 0.01 (Fig. S1A). Among these secretory proteins, ITLN1 is one of the most significantly downregulated and the only adipokine (Table S5). The Cancer Genome Atlas (TCGA) database showed a significant decrease in ITLN1 mRNA levels in the majority of cancer types (Fig. S1B) [13]. Kaplan–Meier analyses based on the TCGA cohort demonstrated a significant association between low ITLN1 expression and unfavorable prognosis in patients with HCC (Fig. S1C). We evaluated ITLN1 expression in paired tumor and adjacent non-tumor tissues from 95 patients in Tongji cohort to validate these findings (Table S1). The ITLN1 mRNA level was markedly downregulated in tumor tissues from patients in Tongji cohort (Fig. 1A and B). Western blot analysis in 18 randomly selected paired tissues from the Tongji cohort further confirmed the reduced ITLN1 protein levels in tumor tissues (Fig. 1C). We further confirmed the downregulation of ITLN1 protein levels in tumor tissues from these 18 patients using immunohistochemistry (Fig. 1D). Kaplan-Meier analysis demonstrated that patients expressing high levels of ITLN1 had significantly longer overall survival in Tongji cohort (Fig. 1E). However, no correlation between ITLN1 expression and recurrence-free survival was found (Fig. S1D). Furthermore, in Tongji cohort, there was a negative correlation between ITLN1 expression and tumor size, as well as the risks of cirrhosis and vascular invasion (Fig. 1F). Nevertheless, no significant correlations were detected between ITLN1 and other clinically relevant parameters, including age, gender, HBsAg status, alpha-fetoprotein (AFP) levels, tumor number, TNM stage, and tumor differentiation (Fig. S1E). Moreover, analysis of the Cancer Cell Line Encyclopedia (CCLE) database indicated generally low ITLN1 expression in HCC cell line (Fig. S1F) [14]. The relative ITLN1 expression level was then verified in several human HCC cell lines (Fig. 1G and H). These collective results emphasize the clinical relevance of ITLN1 as a downregulated secretory adipokine in HCC.

### ITLN1 inhibits the proliferation of HCC cells in vitro

**Fig. 2:**
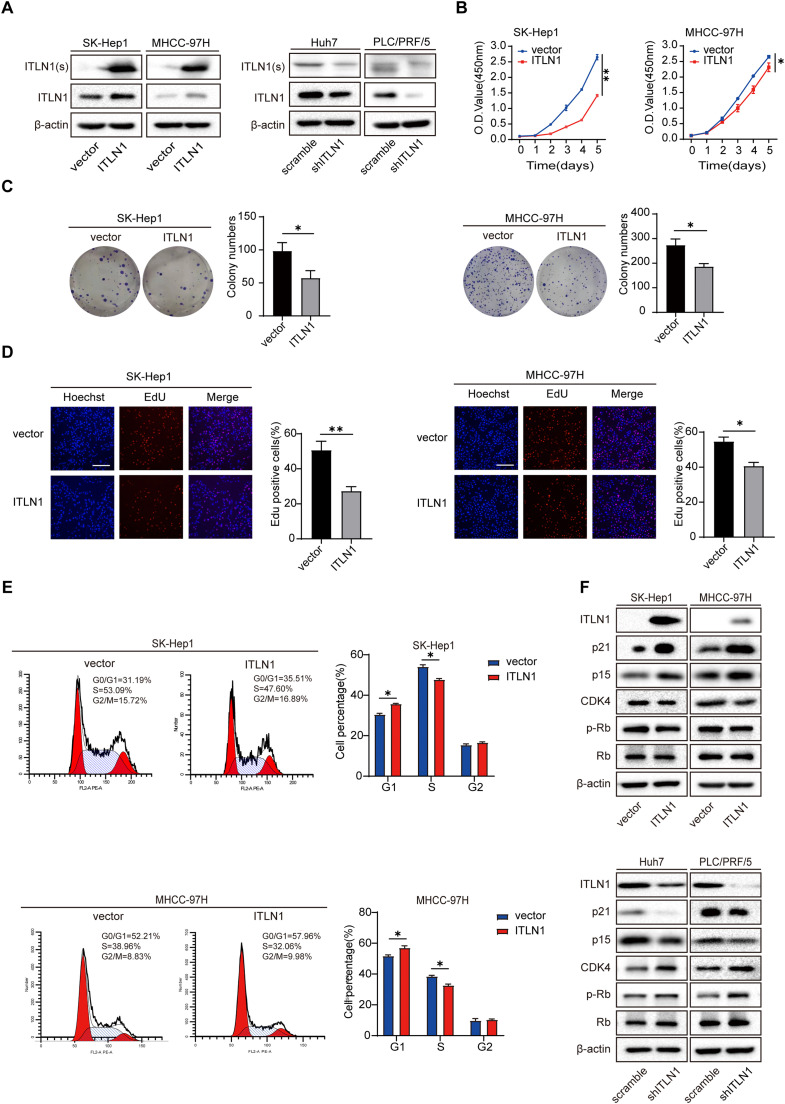
ITLN1 inhibits the proliferation and cell cycle progression of HCC cells in vitro. (A) ITLN1 in the culture supernatant(s) and lysates were detected by western blot. Overexpression of ITLN1 in SK-Hep1 and MHCC-97H cells and knockdown of ITLN1 in Huh7 and PLC/PRF/5 were achieved through lentiviral infection. (B) CCK-8 assay in ITLN1-overexpressed HCC cells. (C) Colony formation assay images and quantification in ITLN1-overexpressed HCC cells. (D) EdU incorporation assay images and quantification of positive cells in ITLN1-overexpressed HCC cells. Scale bars, 100 μm. (E) Cell cycle distribution profiles in ITLN1-overexpressed HCC cells. (F) ITLN1, p21, p15, CDK4, p-Rb, and Rb were detected by western blot in ITLN1-overexpressed and ITLN1-knockdown HCC cells. In (B-E), data are from three independent repetitive experiments and shown as mean ± SD. The statistical significance was detected using the student’s *t*-test. *p < 0.05, **p < 0.01, ***p < 0.001. Abbreviations: O.D., optical density.

Subsequently, we conducted ITLN1 overexpression in SK-Hep1 and MHCC-97H cells, as well as ITLN1 knockdown in Huh7 and PLC/PRF/5 cells to investigate the role of ITLN1 in HCC (Figs. 2A and S2A). Collectively, the results of CCK-8 assay, colony formation assay and EdU assay indicated that overexpression of ITLN1 significantly inhibited the proliferation and colony-forming ability of HCC cells (Fig. 2B–D). Conversely, ITLN1 knockdown promoted the proliferation and colony formation in HCC cells (Fig. S2B–D). However, neither overexpression nor knockdown of ITLN1 influenced the migration potential of the HCC cells (Fig. S3A and B).

### ITLN1 induces cell cycle arrest in the G0/G1 phase

Cell cycle alterations are widely recognized as pivotal factors influencing cell proliferation [15,16]. Cell cycle distribution and related checkpoint factors were studied to evaluate the correlation between cell cycle disturbance and ITLN1-mediated cell growth inhibition. Flow cytometry results revealed that ITLN1 overexpression caused more cells to arrest in the G0/G1 phase of the cell cycle (Fig. 2E), whereas knockdown of ITLN1 had the opposite effect (Fig. S2E). Furthermore, ITLN1 overexpression reduced the protein levels of the critical G1/S transition regulator cyclin-dependent kinase 4 (CDK4), while concurrently elevating the levels of cyclin-dependent kinase inhibitor 2B (p15) and cyclin-dependent kinase inhibitor 1A (p21) (Fig. 2F). However, no discernible difference in p15 and p21 mRNA levels induced by ITLN1 was observed, suggesting that ITLN1 may regulate p15 and p21 expression at a post-transcriptional level. The results suggest that ITLN1 induces cell cycle arrest in the G0/G1 phase in HCC cells.

### ITLN1 inhibits HCC tumor growth in vivo

**Fig. 3:**
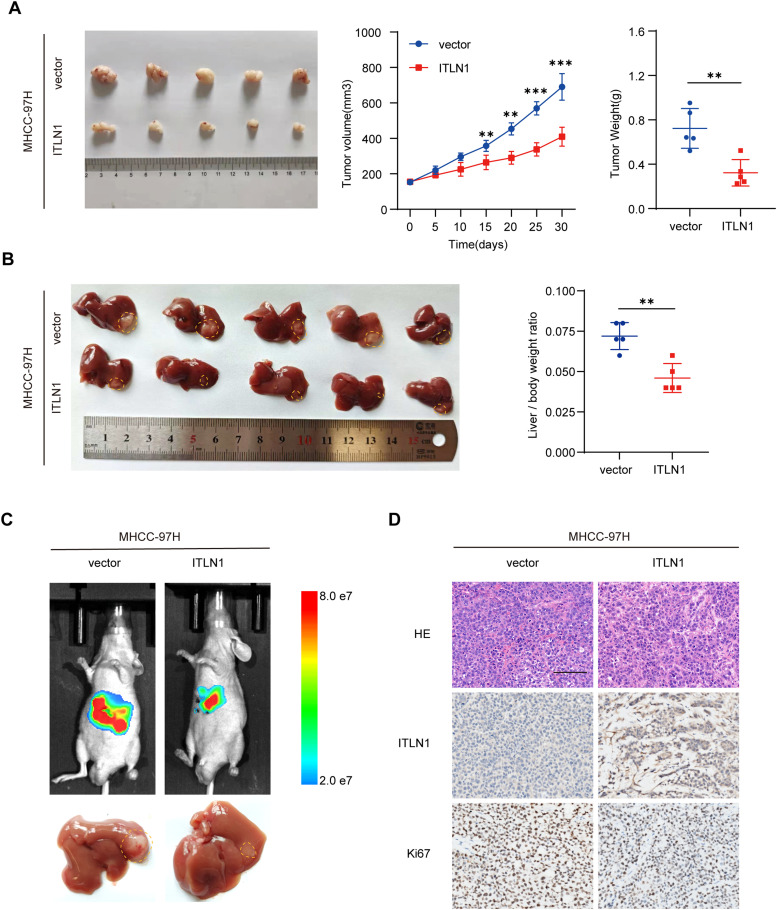
ITLN1 inhibits HCC tumor growth in vivo. (A) Macroscopic views, growth curves, and tumor weights of subcutaneous tumors from nude mice in each group (*n* = 5 mice/group). (B) Macroscopic observation and quantitative analysis of the liver-to-body weight ratio in orthotopic tumors from nude mice across different groups (*n* = 5 mice/group). (C) Representative images of bioluminescence assay and corresponding macroscopic views of orthotopic tumors from nude mice in each group. (D) IHC images of hematoxylin-eosin (H&E), ITLN1, and Ki-67 in the indicated orthotopic tumor groups. Scale bars, 100 μm. In (A and B), data are from three independent repetitive experiments and shown as mean ± SD. The statistical significance was detected using the student’s *t*-test. *p < 0.05, **p < 0.01, ***p < 0.001.

In order to assess the impact of ITLN1 on HCC tumorigenesis in vivo, several animal models were constructed and used. MHCC-97H-ITLN1 and MHCC-97H-vector cells were injected subcutaneously into the nude mice in the experimental and control groups, respectively. Subsequent observations revealed that, compared to the control group, the tumor volume and weight of the experimental group were significantly smaller and lower (Fig. 3A). Further research in an orthotopic xenograft tumor model indicated that ITLN1 overexpression markedly inhibited orthotopic liver tumor growth (Fig. 3B and C). Immunostaining for Ki-67 demonstrated a reduction in staining intensity in orthotopic xenograft tumors with ITLN1 overexpression, confirming that ITLN1 inhibits HCC growth (Fig. 3D). However, there were no differential lung metastases between mice injected with MHCC-97H-ITLN1 or MHCC-97H-vector cells in a lung metastasis model (Fig. S3C). These findings indicate that ITLN1 attenuates HCC growth but has no effect on HCC invasion potential in vivo.

### ERK1/2 signaling modulates the effect of ITLN1 on HCC cell proliferation and cell cycle progression

**Fig. 4:**
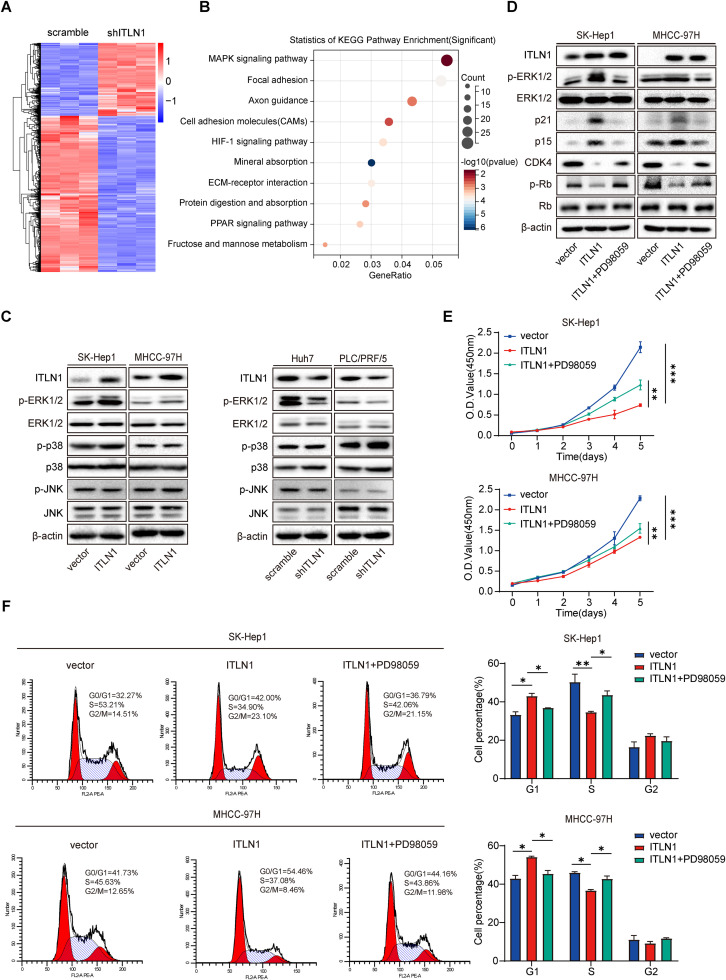
ERK1/2 signaling modulates the effect of ITLN1 on HCC cell growth and cell cycle progression. (A) Heatmap of the differentially expressed genes between ITLN1-scramble and ITLN1-knockdown Huh7 cells (*n* = 3 samples/group). (B) KEGG enrichment analysis and top ten enriched pathways of the ITLN1-regulated genes (*n* = 3 samples/group). (C) Expression levels of MAPK signaling pathway proteins detected by western blot in ITLN1-overexpressed and ITLN1-knockdown HCC cells. (D) Inactivation of ERK1/2 in ITLN1-overexpressed cells reversed p21 upregulation and downstream activating signaling. Cells were treated with the MEK1/2 inhibitor PD98059 (50 μM) for 24 h before harvest and western blot analysis. (E) Viability of HCC cells measured by CCK-8 assay at indicated time point. (F) Cell cycle distribution in each group detected by flow cytometry. Data are from three independent repetitive experiments and shown as mean ± SD. The statistical significance was detected using the student’s *t*-test. *p < 0.05, **p < 0.01, ***p < 0.001.

To unravel the potential mechanism of ITLN1 suppressing HCC cell proliferation, transcriptome profiling on ITLN1-knockdown Huh7 cells was performed (Fig. 4A). RNA sequencing analyses revealed that ITLN1 knockdown in Huh7 cells induced transcriptional changes in 1645 human genes, comprising 1121 upregulated and 524 downregulated genes. Kyoto Encyclopedia of Genes and Genomes (KEGG) pathway enrichment and gene ontology analyses of the differentially expressed genes identified the enrichment of mitogen-activated protein kinase (MAPK) signaling pathway, which may be involved in the negative regulation of cell growth (Figs. 4B and S4A). Furthermore, gene set enrichment analysis (GSEA) showed that enriched MAPK signaling and ITLN1 expression was positively correlated, with ITLN1 being implicated in arresting cell cycle (Fig. S4B and C) [17,18]. The MAPK signaling has been widely proven to have essential roles in regulating various cell biological processes, including proliferation, development, and cell cycle [19,20]. Hence, we opted to delve deeper into the MAPK signaling pathway and investigate the impact of ITLN1 expression on the activation of ERK1/2, JNK, and p38 in HCC cells. Our study revealed that ITLN1 overexpression increased p-ERK1/2 protein levels, whereas knockdown of ITLN1 decreased p-ERK1/2 expression (Fig. 4C). However, there was no difference in the activation level of JNK and p38 in both the ITLN1-overexpressed and ITLN1-knockdown HCC cells when compared to control groups. Consistently, IHC staining revealed that the protein level of p-ERK1/2 was positively correlated with ITLN1 in subcutaneous tumors (Fig. S4D). Previous studies have substantiated the involvement of the ERK1/2 signaling pathway in regulating p15 and p21 expression, thereby suppressing HCC progression [21,22]. Zhang and colleagues also observed that HPCAL1 suppresses HCC growth by activating the ERK1/2 signaling pathway, thereby enhancing the stability of p21 [23]. In our study, we treated ITLN1-overexpressed HCC cells with 50 μM MEK1/2 inhibitor, PD98059, to investigate the impact of the ERK1/2 signaling on the growth inhibition and regulation of cell cycle-related proteins induced by ITLN1 in HCC cells. We found that PD98059 almost completely abrogated the phosphorylation of ERK1/2 and brought the protein expression of p15, p21, CDK4, and p-Rb to similar levels as the control groups (Fig. 4D). Based on the observation in CCK-8 assay, colony formation assay, EdU assay, and cell cycle distribution, we concluded that PD98059 effectively counteracted the suppressing impact of ITLN1 on HCC cell proliferation and cell cycle progression (Fig. 4E and F and S5A and B). These results indicate that ITLN1 hinders the proliferation of HCC cells via modulation of the ERK1/2 signaling pathway.

### IRF1 facilitates ITLN1 promoter activity

**Fig. 5:**
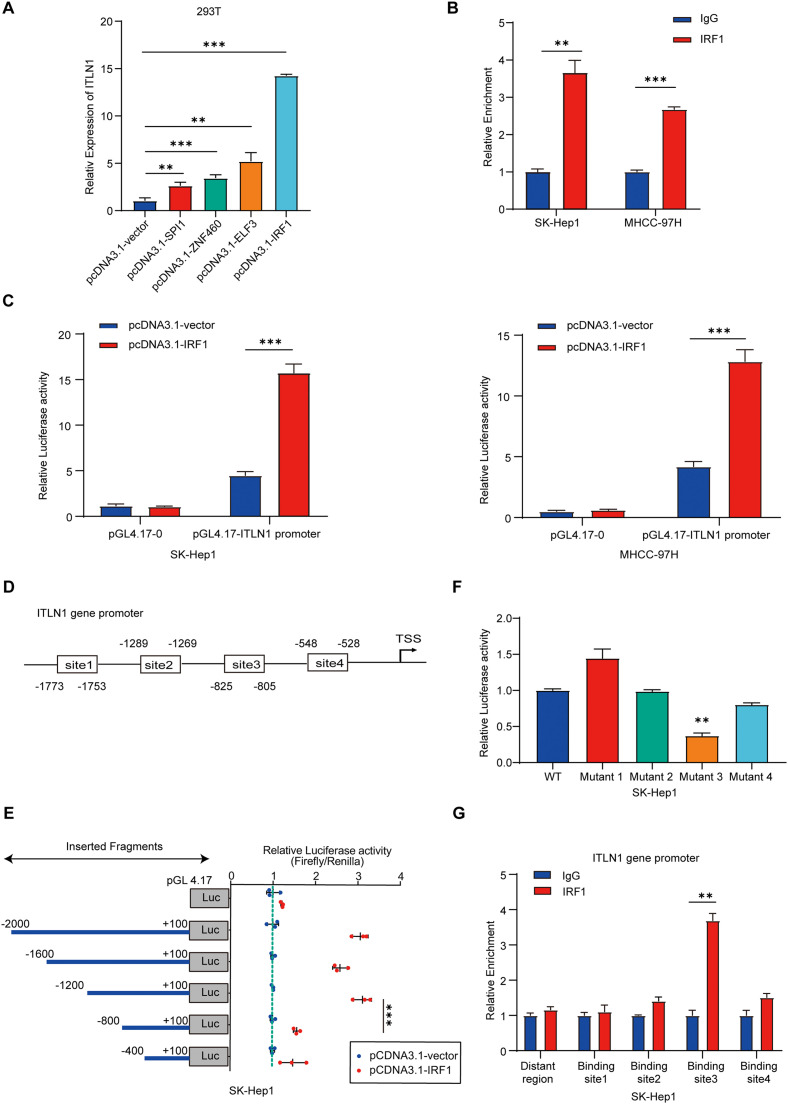
IRF1 facilitates ITLN1 promoter activity. (A) qRT-PCR detecting the mRNA level of ITLN1 after ectopic expression of indicated genes in 293T cells. (B) The binding between IRF1 and ITLN1 promoter was determined by ChIP assay. (C) Ectopic expression of IRF1 in HCC cells and detection of the promoter activity of ITLN1 by dual-luciferase assay. (D) Schematic illustration of the 4 putative IRF1-binding sites in ITLN1 promoter region (site1: CTCTATTTTTTTTTTTTTTTT; site2: TTTTTTTTGCAGTTTTTCTTC; site3: GTTTTGTATCTCTTTCCCCTC; site4: TTTTACTTTTATTTTTAGTAG). (E) Overview of the serial 400-promoter truncations sequence upstream of the ITLN1 transcription start site (left) and their corresponding transcriptional activities (right). (F) Transcriptional activity of luciferase reporter construct containing WT or mutant IRF1-binding sites was determined by a dual-luciferase assay. (G) ChIP assay analysis of the binding between IRF1 and putative binding sites in the ITLN1 promoter. In (A-C and E-G), data are from three independent repetitive experiments and shown as mean ± SD. The statistical significance was detected using the student’s *t*-test. *p < 0.05, **p < 0.01, ***p < 0.001. Abbreviations: WT, wild-type.

Until now, no study has elucidated the epigenetic regulatory mechanism of ITLN1 in tumors. Therefore, we proceeded to predict potential transcription factors of ITLN1 utilizing the JASPAR database (Table S6) [24]. Several possible transcription factors targeting the ITLN1 promoter sequence were then overexpressed in 293T cells by transient transfection (Fig. 5A). Among these transcription factors, IRF1 was most capable of positively regulating ITLN1 expression. The ChIP assay showed that the IRF1-targeting ITLN1 promoter regions were immunoprecipitated by the antibody specific to IRF1 when compared to an unspecific antibody (isotype IgG) in cultured HCC cells (Fig. 5B). In addition, the luciferase reporter assay revealed that ectopic expression of IRF1 increased the ITLN1 transcriptional activity (Fig. 5C). Subsequently, through analysis of the bioinformatics databases JASPAR and hTFtarget, we identified four potential IRF1-binding sites within the promoter region of ITLN1 (Fig. 5D)[25]. Truncation and mutation assays showed that the third IRF1-binding site (site3, −825 to −805) was critical for enhancing the promoter activity of ITLN1 (Fig. 5E and F). The ChIP assay further validated that the site3 sequence co-precipitated with endogenous IRF1 in HCC cells (Fig. 5G). Collectively, all results indicate that IRF1 enhances ITLN1 promoter activity in HCC cells.

### IFNγ-IRF1-ITLN1 axis in HCC

**Fig. 6:**
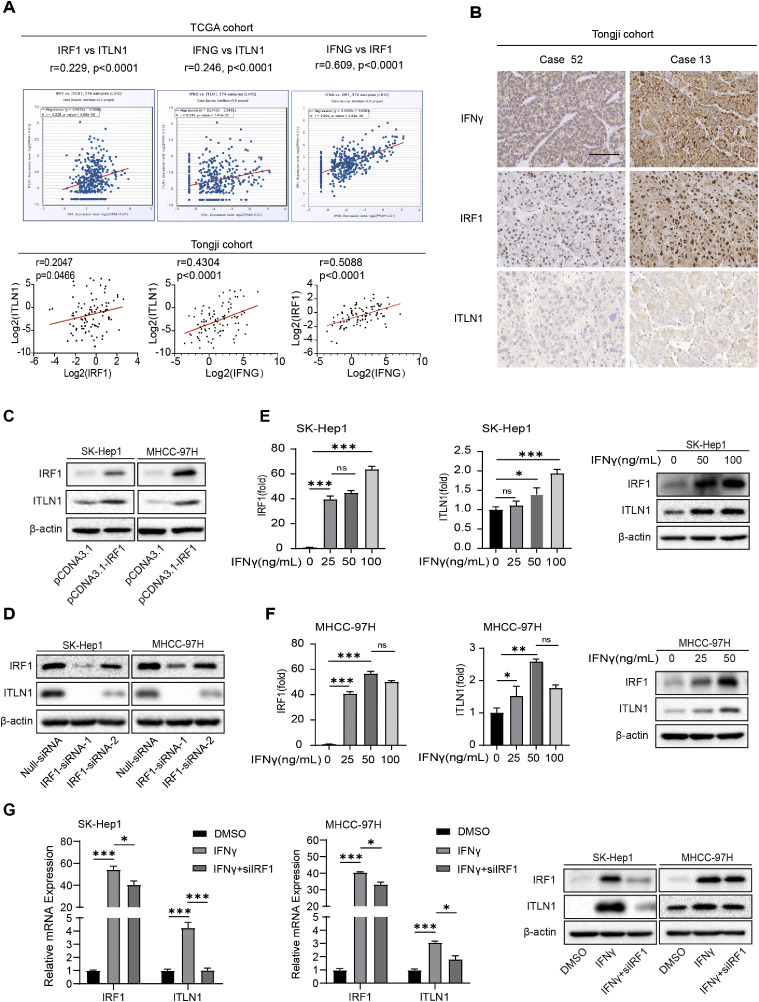
IFNγ-IRF1-ITLN1 axis in HCC. (A) Analysis of the mRNA expression correlation among ITLN1, IRF1, and IFNγ in HCC patients from the TCGA database and Tongji cohort. (B) Immunohistochemical staining to assess the correlation between protein expressions of ITLN1, IRF1, and IFNγ in the Tongji cohort. Representative examples of the staining are provided. Scale bars, 100 μm. (C, D) Western blot analysis of ITLN1 and IRF1 expression in SK-Hep1 and MHCC-97H cells transfected with pcDNA3.1-IRF1 plasmids or IRF1 siRNA for 48 h. (E, F) Detection of ITLN1 and IRF1 expression levels in HCC cells using qRT-PCR and western blot. Cells were incubated with different concentrations of IFNγ for 48 h before harvest. (G) The expression levels of ITLN1 and IRF1 were determined by qRT-PCR and western blot in HCC cells. SK-Hep1 cells were incubated with 100 ng/mL of IFNγ and MHCC-97H cells were incubated with 50 ng/mL of IFNγ for 12 h. Then, IRF1 siRNA was added to the medium for further culture for 36 h. In (E-G), data are from three independent repetitive experiments and shown as mean ± SD. The statistical significance was detected using the student’s *t*-test. *p < 0.05, **p < 0.01, ***p < 0.001. Abbreviations: siRNA, small interference RNA; ns, no significant difference.

We investigated the correlation between ITLN1, IRF1, and IFNγ, a classic upstream cytokine of IRF1 in HCC tissues from the TCGA and Tongji cohorts. qRT-PCR and IHC analysis of the HCC tissues repeatedly confirmed that ITLN1 was positively correlated with IRF1 and IFNγ (Fig. 6A and B). IRF1 overexpression facilitated the expression of ITLN1, whereas knockdown of IRF1 had the opposite effect (Fig 6C and D and S6A and B). Additionally, both the expression levels of IRF1 and ITLN1 increased with escalating concentrations of IFNγ (Fig. 6E and F). Furthermore, IRF1 knockdown attenuated the IFNγ-induced upregulation of ITLN1, indicating that IRF1 mediates the regulatory effect of IFNγ on ITLN1 (Fig. 6G). These findings suggest that there exists an IFNγ-IRF1-ITLN1 axis in HCC.

### Involvement of ITLN1 in mediating the IRF1-induced suppression of HCC cell proliferation and cell cycle progression

**Fig. 7:**
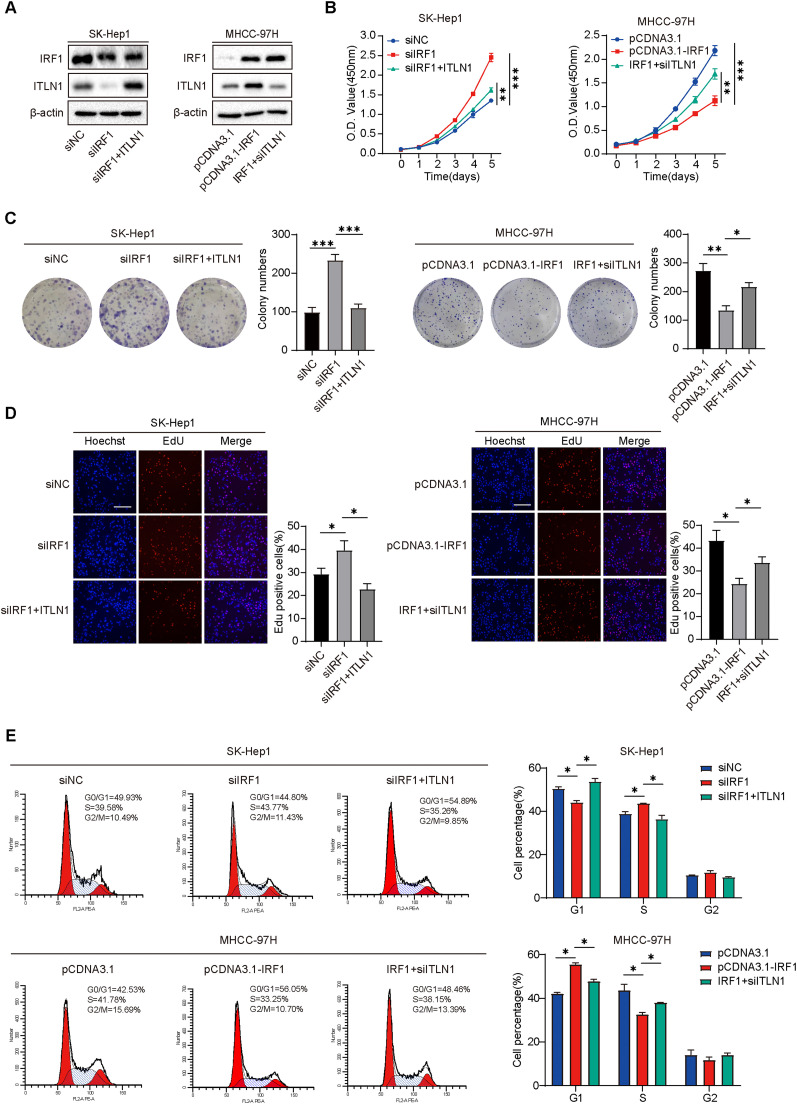
Involvement of ITLN1 in mediating the IRF1-induced suppression of HCC cell proliferation and cell cycle progression. SK-Hep1 cells were incubated with IRF1 siRNA for 12 h and transfected with pcDNA-ITLN1 for another 36 h culture before harvest. MHCC-97H cells were transfected with pcDNA-IRF1 plasmids for 12 h and then incubated with ITLN1 siRNA for 36 h before harvest. (A) The protein levels of ITLN1 and IRF1 were detected by western blot in SK-Hep1 and MCHH-97H cells. (B) CCK-8 assay in SK-Hep1 and MHCC-97H cells with different pretreatment. (C) Representative images of colony formation assay and quantification of colony numbers in SK-Hep1 and MHCC-97H cells with different pretreatment. (D) Representative images of EdU incorporation assay and quantification of positive EdU staining cells in SK-Hep1 and MHCC-97H cells with different pretreatment. Scale bars, 100 μm. (E) Cell cycle distribution profiles in the indicated cells. In (B-E), data are from three independent repetitive experiments and shown as mean ± SD. The statistical significance was detected using the student’s *t*-test. *p < 0.05, **p < 0.01, ***p < 0.001. Abbreviations: O.D., optical density; siRNA, small interfering RNA.

While numerous studies on IRF1 have primarily focused on its effects on tumor invasion and the immune microenvironment [26,27], we conducted a "rescue" experiment to evaluate the correlation between IRF1 and HCC proliferation, specifically investigating the involvement of ITLN1 in this regulatory process. We overexpressed ITLN1 in IRF1-knockdown SK-Hep1 cells and knocked down ITLN1 in IRF1-overexpressed MHCC-97H cells (Fig. 7A). Subsequent assays collectively suggest that IRF1 overexpression suppresses HCC cell proliferation, while the knockdown of IRF1 promotes HCC cell proliferation. The overexpression and knockdown of ITLN1 partially restored the proliferative abilities of IRF1-knockdown SK-Hep1 cells and IRF1-overexpressed MHCC-97H cells, respectively (Fig. 7B–D). Meanwhile, the flow cytometry revealed that ectopic expression of ITLN1 led to more IRF1-knockdown SK-Hep1 cells being arrested in the G0/G1 phase (Fig. 7E). Moreover, knockdown of ITLN1 restored the inhibitory effects caused by the ectopic expression of IRF1 on G1/S transition in MHCC-97H cells to some degree (Fig. 7E). These results underscore the active involvement of ITLN1 in the process through which IRF1 inhibits HCC cell proliferation and cell cycle progression.

### IFNγ inhibits HCC cell proliferation and cell cycle progression partly through ITLN1

**Fig. 8:**
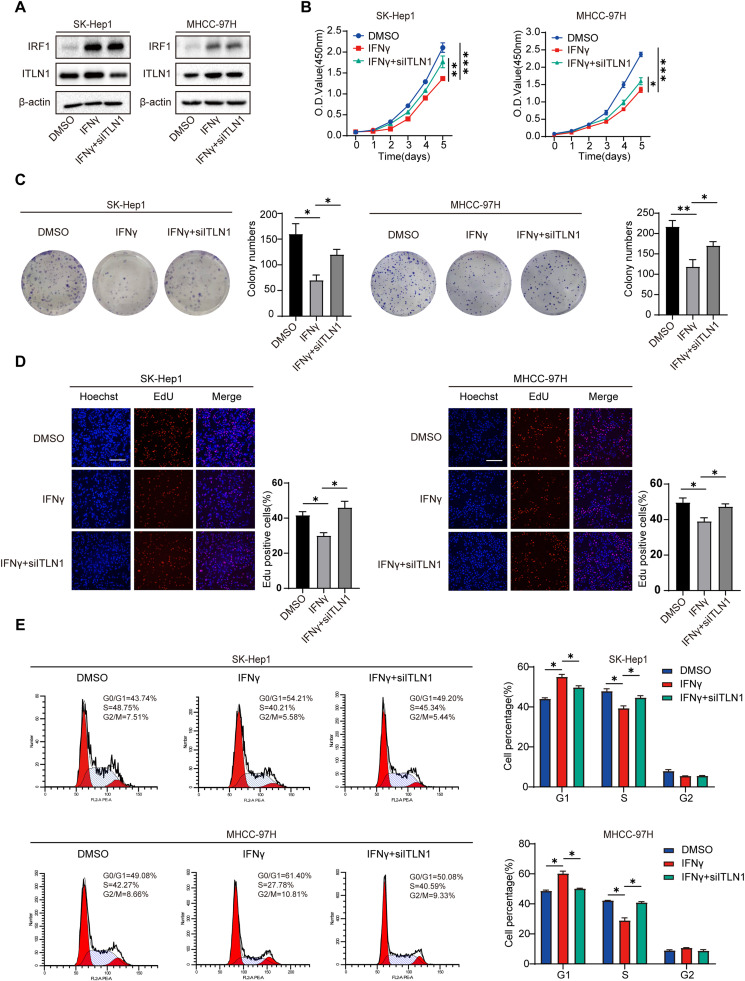
IFNγ inhibits HCC cell proliferation and cell cycle progression partly through ITLN1. SK-Hep1 cells were incubated with 100ng/mL IFNγ and MHCC-97H cells were incubated with 50 ng/mL IFNγ for 12 h. ITLN1 siRNA was then added to the medium for further culture for 36 h. (A) The protein levels of ITLN1 and IRF1 were detected by western blot in SK-Hep1 and MCHH-97H cells. (B) CCK-8 assay in SK-Hep1 and MHCC-97H cells with different pretreatment. (C) Representative images of colony formation assay and quantification of colony numbers in SK-Hep1 and MHCC-97H cells with different pretreatment. (D) Representative images of EdU incorporation assay and quantification of positive EdU staining cells in SK-Hep1 and MHCC-97H cells with different pretreatment. Scale bars, 100 μm. (E) Cell cycle distribution profiles in the indicated cells. In (B-E), data are from three independent repetitive experiments and shown as mean ± SD. The statistical significance was detected using the student’s *t*-test. *p < 0.05, **p < 0.01, ***p < 0.001. Abbreviations: O.D., optical density; siRNA, small interfering RNA.

Subsequently, we explored the potential involvement of ITLN1 in the IFNγ-mediated suppression of HCC proliferation, a phenomenon observed in HCC and several other tumors [[28], [29], [30], [31]]. SK-Hep1 and MHCC-97H cells were incubated with IFNγ at a final concentration of 100ng/mL and 50ng/mL, respectively for 12 h. Subsequently, siRNAs targeting ITLN1 were introduced to the culture medium for an additional 36 h. We then assessed the protein levels of IRF1 and ITLN1 through western blot analysis (Fig. 8A). Our research showed that IFNγ suppressed HCC cell proliferation, while subsequent knockdown of ITLN1 compromised this inhibitory effect on the HCC cells (Fig. 8B–D). The analysis of the cell cycle revealed that IFNγ induced cell cycle arrest, while the ITLN1 knockdown partially reversed this inhibitory effect of IFNγ on HCC (Fig. 8E). These findings suggest that IFNγ inhibits HCC cell proliferation and cell cycle progression, at least in part, through ITLN1.

## Discussion

Given the complex biological characteristics, high malignancy, and unfavorable prognosis associated with HCC, additional promising targets are urgently sought to enhance clinical efficacy. Previous research has demonstrated that several adipokines have a critical effect on HCC initiation and progression [[32], [33], [34]]. Hence, more in-depth research on adipokines may help in the management of HCC.

ITLN1 is a newly discovered adipokine whose function in cancer development remains unclear. ITLN1 has been reported to inhibit metastasis in neuroblastoma, gastric tumor, and ovarian cancer [8,9,35]. Here, we identified ITLN1 as an emerging prognostic indicator for individuals with HCC through RNA microarray analysis. We observed a significant downregulation of ITLN1 in HCC. The decreased expression of ITLN1 is in conjunction with an unfavorable outlook for patients, suggesting a potential tumor-suppressive role in HCC. Our results regarding the correlation between ITLN1 and cirrhosis, vascular invasion, and tumor size provided clinical evidence of the correlation between ITLN1 and HCC progression, although the strength of this evidence may need to be further validated in more HCC cohorts. Through gain-of-function and loss-of-function experiments, we elucidated that ITLN1 suppresses the proliferation of HCC. However, the invasive potential of HCC cells was not affected by either overexpression or knockdown of ITLN1, which is in alignment with the discoveries made by Zhang et al. [35]. Through further experimental verification, we demonstrated that ectopic expression of ITLN1 induced cell cycle arrest while upregulating p15 and p21 expression. These results indicate that ITLN1 attenuates HCC cell proliferation in part through cell cycle control. Additionally, we observed that ITLN1 elevated protein levels, but not mRNA levels, suggesting that ITLN1 may regulate p15 and p21 expression at a post-transcriptional level. The degradation of many proteins, including p21, is regulated through the proteasome-mediated turnover process [[36], [37], [38]]. Meanwhile, transcriptome profiling of ITLN1-knockdown Huh7 cells showed the enrichment of MAPK signaling. Subsequently, western blot detection revealed that ERK1/2, but not JNK or p38 signaling was activated. Furthermore, it has been demonstrated that the activation of ERK1/2 signaling pathway in HCC stabilizes the p21 protein by inhibiting p21 ubiquitination degradation, thereby inhibiting HCC cell proliferation [22,23]. It has also been reported that the activating ERK1/2 signaling results in an upregulation of p15 expression in HCC [21]. So, we speculated that ITLN1 upregulates p15 and p21 expression by activating the ERK1/2 signaling pathway. Pretreatment with PD98059, an inhibitor of MEK1/2, the upstream kinase of ERK1/2 for 24 h, effectively prevented ITLN1-induced cell cycle arrest and resulted in a 50–75 % decrease of p15 and p21 proteins. These consistent findings, in line with prior studies, suggest that ITLN1 inhibits the proliferation of HCC cells, at least to some extent, by activating the ERK1/2 signaling pathway (Fig. S7).

Although ITLN1 overexpression led to only a modest increase in the proportion of tumor cells in the G0/G1 phase, the marked reduction in proliferation suggests that additional mechanisms may be involved. For instance, ITLN1 has been reported to promote tumor cell apoptosis in vitro via modulation of the PI3K/Akt pathway and Sirt1-dependent p53 deacetylation [35,39]. Moreover, in previous studies on non-alcoholic fatty liver disease, ITLN1 was shown to preserve protective autophagy through normalization of AMPKα/mTOR signaling, thereby alleviating cellular stress and lipid accumulation [40]. In the context of HCC, ITLN1 may similarly contribute to growth suppression and metabolic stabilization through this pathway, which merits further investigation. Collectively, these findings imply that ITLN1 may exert multifaceted effects on HCC cell proliferation and survival through the coordinated regulation of cell cycle, apoptosis, autophagy, and possibly other pathways yet to be elucidated. Future studies are warranted to delineate these interactions in detail.

IRF1, a classical downstream target of IFNγ, functions as a transcription factor critical in mediating IFNγ-induced anti-proliferation, apoptosis, invasion inhibition, and antitumor immunity in cancer [[41], [42], [43], [44], [45]]. Over the past few years, the role of the IFNγ-IRF1 axis in HCC has been receiving increasing attention. Yan et al. reported that IFNγ plays its role in inhibiting growth and promoting cell death by inducing autophagy through IRF1 in HCC [46]. Moreover, the IRF1-dependent upregulation of PD-L1 expression triggered by IFNγ provides new insights into immune checkpoint blockade therapy in HCC [44]. However, many mechanisms of the IFNγ-IRF1 axis in HCC remain to be described due to relatively few studies. Our findings further support an anti-tumorigenic role for the IFNγ-IRF1 axis in HCC. A thorough analysis of both the TCGA and our local patient databases uncovered a decrease in IFNγ and IRF1 expression in HCC, with a positive correlation observed with ITLN1 expression. This discovery prompted us to delve deeper into the investigation and analysis of the role of the IFNγ-IRF1 axis in ITLN1 expression. Subsequently, we found that IFNγ promotes the expression of ITLN1 through IRF1 in a dose-dependent manner. Our further investigation identified the existence of putative binding sites for IRF1 on the ITLN1 promoter. Through mining public databases including JASPAR and hTFtarget and performing ChIP and luciferase activity assays, we proceeded to identify and determine the binding sequence. Subsequently, we verified the profoundly inhibitory role of the IFNγ-IRF1-ITLN1 axis in HCC cell proliferation. This finding provides additional insights into an alternative pathway by which p21 participated in the IFNγ-IRF1 axis-induced dose-dependent G0/G1 arrest in previous studies [47,48]. To our knowledge, this study is pioneering in uncovering the IFNγ-IRF1-ITLN1 axis in cancer, and its function and mechanism need to be further studied. Also, it is interesting to explore how IFNγ-IRF1-ITLN1 axis-mediated immune and metabolic changes affect the HCC tumor microenvironment.

Not surprisingly, this study possesses certain limitations. Firstly, the clinical sample size was relatively small and a more comprehensive and extended clinical follow-up is needed. Secondly, we attempted but failed to identify any membrane receptor of ITLN1 on HCC cells, and we did not establish precisely the receptor and pathway through which ITLN1 regulates ERK1/2 signaling. The ITLN1 receptor has not been reported in previous studies, and this will be one of our future major research directions. Thirdly, being limited by scientific research funds and experimental technologies, we have not completed the application of purified ITLN1 protein in animal models, which makes the article lack more advanced evidence supporting the therapeutic value of ITLN1 in HCC. Last but equally important, hepatokines are a fascinating class of secretory molecules with the potential to influence other cells in the microenvironment. However, in this study, we did not investigate the hepatokine function of ITLN1 in the tumor microenvironment. Additionally, while IFNγ/IRF1 regulates genes related to the immune system, the role of ITLN1 in modulating the activity or status of other immune cells in HCC remains unanswered. These aspects represent interesting avenues for future research.

To sum up, our study identified ITLN1 as a new prognostic biomarker for HCC, uncovering the antagonistic impact on HCC growth through the mediation of ERK1/2 signaling activation. Meanwhile, we have revealed the possible transcriptional regulation mechanism of the IFNγ-IRF1-ITLN1 axis for the first time and verified some functions of this axis in HCC. This research offers fresh perspectives on exploring the interplay between adipokines and HCC, potentially paving the way for developing novel anticancer agents.

## Funding statement

This study was supported by the National Natural Science Foundation of China (no 81902839), the Non-profit Central Research Institute Fund of Chinese Academy of Medical Sciences (2020-PT330-003) and the Open Research Fund of NHC Key Laboratory of Prevention and Treatment of Central Asia High Incidence Diseases.

## Ethics statement

The ethics committee of Tongji Hospital approved this study. All procedures in the animal experiment were conducted in accordance with the Guide for the Care and Use of Laboratory Animals.

## CRediT authorship contribution statement

**Tong Yuan:** Writing – original draft, Methodology, Investigation, Conceptualization. **Junjie Liu:** Methodology, Investigation, Data curation. **Ronghua Zhu:** Methodology, Investigation, Formal analysis. **Jiang Li:** Methodology, Investigation. **Zhiyong Huang:** Methodology, Investigation. **Huifang Liang:** Methodology, Investigation. **Haisu Tao:** Writing – review & editing, Funding acquisition. **Erlei Zhang:** Writing – review & editing, Funding acquisition, Conceptualization.

## Declaration of competing interest

The authors declare that they have no known competing financial interests or personal relationships that could have appeared to influence the work reported in this paper.

## Data Availability

The datasets generated and analyzed for the current study are available from the corresponding author upon reasonable request.

## Appendix Supplementary materials

Image, image 1

Image, image 2

Image, image 3

Image, image 4

Image, image 5

Image, image 6

Image, image 7

Image, application 8

Image, application 9

Image, application 10

Image, application 11

Supporting information Image, application 12

## References

[1] Huang D.Q., El-Serag H.B., Loomba R. (2021). Global epidemiology of NAFLD-related HCC: trends, predictions, risk factors and prevention. Nat. Rev. Gastroenterol. Hepatol..

[2] Polyzos S.A., Kountouras J., Mantzoros C.S. (2016). Adipokines in nonalcoholic fatty liver disease. Metabolism.

[3] Stojsavljević S., Gomerčić Palčić M., Virović Jukić L., Smirčić Duvnjak L., Duvnjak M. (2014). Adipokines and proinflammatory cytokines, the key mediators in the pathogenesis of nonalcoholic fatty liver disease. World J. Gastroenterol..

[4] Nagaraju G.P., Aliya S., Alese O.B. (2015). Role of adiponectin in obesity related gastrointestinal carcinogenesis. Cytok. Grow. Fact. Rev..

[5] Paval D.R., Di Virgilio T.G., Skipworth R.J.E., Gallagher I.J. (2022). The emerging role of intelectin-1 in cancer. Front. Oncol..

[6] Chen L., Jin X.H., Luo J. (2021). ITLN1 inhibits tumor neovascularization and myeloid derived suppressor cells accumulation in colorectal carcinoma. Oncogene.

[7] Au-Yeung C.L., Yeung T.L., Achreja A. (2020). ITLN1 modulates invasive potential and metabolic reprogramming of ovarian cancer cells in omental microenvironment. Nat. Commun..

[8] Li D., Zhao X., Xiao Y. (2015). Intelectin 1 suppresses tumor progression and is associated with improved survival in gastric cancer. Oncotarget.

[9] Li D., Mei H., Pu J. (2015). Intelectin 1 suppresses the growth, invasion and metastasis of neuroblastoma cells through up-regulation of N-myc downstream regulated gene 2. Mol. Cancer.

[10] Li J., Tao H.S., Yuan T., Huang Z.Y., Zhang E.L. (2023). Intelectin-1 is a novel prognostic biomarker for hepatocellular carcinoma. Med. (Baltim.).

[11] Huang Z., Chu L., Liang J. (2021). H19 Promotes HCC Bone Metastasis Through Reducing Osteoprotegerin Expression in a Protein Phosphatase 1 Catalytic Subunit Alpha/p38 Mitogen-Activated Protein Kinase-Dependent Manner and Sponging microRNA 200b-3p. Hepatology.

[12] Liang J., Li G., Liao J. (2022). Non-coding small nucleolar RNA SNORD17 promotes the progression of hepatocellular carcinoma through a positive feedback loop upon p53 inactivation. Cell Death. Differ..

[13] Blum A., Wang P., Zenklusen J.C. (2018). SnapShot: TCGA-analyzed tumors. Cell.

[14] Barretina J., Caponigro G., Stransky N. (2012). The Cancer Cell Line Encyclopedia enables predictive modelling of anticancer drug sensitivity. Nature.

[15] Coffman J.A. (2004). Cell cycle development. Dev. Cell.

[16] Zatulovskiy E., Zhang S., Berenson D.F., Topacio B.R., Skotheim J.M. (2020). Cell growth dilutes the cell cycle inhibitor Rb to trigger cell division. Science (1979).

[17] Subramanian A., Tamayo P., Mootha V.K. (2005). Gene set enrichment analysis: a knowledge-based approach for interpreting genome-wide expression profiles. Proc. Natl. Acad. Sci. U.S.A..

[18] Liberzon A., Birger C., Thorvaldsdóttir H., Ghandi M., Mesirov J.P., Tamayo P. (2015). The Molecular Signatures Database (MSigDB) hallmark gene set collection. Cell Syst..

[19] Wu P.K., Becker A., Park J.I. (2020). Growth Inhibitory Signaling of the Raf/MEK/ERK Pathway. Int. J. Mol. Sci..

[20] Lavoie H., Gagnon J., Therrien M. (2020). ERK signalling: a master regulator of cell behaviour, life and fate. Nat. Rev. Mol. Cell Biol..

[21] Wen-Sheng W. (2003). ERK signaling pathway is involved in p15INK4b/p16INK4a expression and HepG2 growth inhibition triggered by TPA and Saikosaponin a. Oncogene.

[22] Chen T., Huang H., Zhou Y. (2018). HJURP promotes hepatocellular carcinoma proliferation by destabilizing p21 via the MAPK/ERK1/2 and AKT/GSK3β signaling pathways. J. Exp. Clin. Cancer Res..

[23] Zhang Y., Liu Y., Duan J. (2016). Hippocalcin-like 1 suppresses hepatocellular carcinoma progression by promoting p21(Waf/Cip1) stabilization by activating the ERK1/2-MAPK pathway. Hepatology.

[24] Fornes O., Castro-Mondragon J.A., Khan A. (2020). JASPAR 2020: update of the open-access database of transcription factor binding profiles. Nucl. Acid. Res..

[25] Zhang Q., Liu W., Zhang H.M. (2020). hTFtarget: a comprehensive database for regulations of human transcription factors and their targets. Genom. Proteom. Bioinformat..

[26] Taniguchi T., Ogasawara K., Takaoka A., Tanaka N. (2001). IRF family of transcription factors as regulators of host defense. Annu. Rev. Immunol..

[27] Tanaka N., Taniguchi T. (2000). The interferon regulatory factors and oncogenesis. Semin. Cancer Biol..

[28] Guinn Z.P., Petro T.M. (2018). IFN-γ synergism with poly I:c reduces growth of murine and human cancer cells with simultaneous changes in cell cycle and immune checkpoint proteins. Cancer Lett..

[29] Guan Y.Q., Li Z., Yang A. (2012). Cell cycle arrest and apoptosis of OVCAR-3 and MCF-7 cells induced by co-immobilized TNF-α plus IFN-γ on polystyrene and the role of p53 activation. Biomaterials.

[30] Matsushita H., Hosoi A., Ueha S. (2015). Cytotoxic T lymphocytes block tumor growth both by lytic activity and IFNγ-dependent cell-cycle arrest. Cancer Immunol. Res..

[31] Kong R., Wang N., Han W., Bao W., Lu J. (2021). IFNγ-mediated repression of system xc(-) drives vulnerability to induced ferroptosis in hepatocellular carcinoma cells. J. Leukoc. Biol..

[32] Kucukoglu O., Sowa J.P., Mazzolini G.D., Syn W.K., Canbay A. (2021). Hepatokines and adipokines in NASH-related hepatocellular carcinoma. J. Hepatol..

[33] Karagozian R., Derdák Z., Baffy G. (2014). Obesity-associated mechanisms of hepatocarcinogenesis. Metabolism.

[34] Divella R., Mazzocca A., Daniele A., Sabbà C., Paradiso A. (2019). Obesity, nonalcoholic fatty liver disease and adipocytokines network in promotion of cancer. Int. J. Biol. Sci..

[35] Zhang Y.Y., Zhou L.M. (2013). Omentin-1, a new adipokine, promotes apoptosis through regulating Sirt1-dependent p53 deacetylation in hepatocellular carcinoma cells. Eur. J. Pharmacol..

[36] Rousseau A., Bertolotti A. (2018). Regulation of proteasome assembly and activity in health and disease. Nat. Rev. Mol. Cell Biol..

[37] Mishra R., Upadhyay A., Prajapati V.K., Mishra A. (2018). Proteasome-mediated proteostasis: novel medicinal and pharmacological strategies for diseases. Med. Res. Rev..

[38] Bloom J., Amador V., Bartolini F., DeMartino G., Pagano M. (2003). Proteasome-mediated degradation of p21 via N-terminal ubiquitinylation. Cell.

[39] Ji H., Wan L., Zhang Q., Chen M., Zhao X. (2019). The effect of omentin-1 on the proliferation and apoptosis of colon cancer stem cells and the potential mechanism. J. BUON: Off. J. Balkan Union Oncol..

[40] Huang Z., Luo L., Xiao Z., Xiong M., Wen Z. (2024). Omentin-1 mitigates non-alcoholic fatty liver disease by preserving autophagy through AMPKα/mTOR signaling pathway. Sci. Rep..

[41] Nicolini A., Carpi A., Rossi G. (2006). Cytokines in breast cancer. Cytokine Grow. Fact. Rev..

[42] Zhou F. (2009). Molecular mechanisms of IFN-gamma to up-regulate MHC class I antigen processing and presentation. Int. Rev. Immunol..

[43] Jiang M., Jia K., Wang L. (2021). Alterations of DNA damage response pathway: biomarker and therapeutic strategy for cancer immunotherapy. Acta Pharm. Sin. B.

[44] Yan Y., Zheng L., Du Q., Yan B., Geller D.A. (2020). Interferon regulatory factor 1 (IRF-1) and IRF-2 regulate PD-L1 expression in hepatocellular carcinoma (HCC) cells. Cancer Immunol. Immunther..

[45] Camicia R., Bachmann S.B., Winkler H.C. (2013). BAL1/ARTD9 represses the anti-proliferative and pro-apoptotic IFNγ-STAT1-IRF1-p53 axis in diffuse large B-cell lymphoma. J. Cell Sci..

[46] Li P., Du Q., Cao Z. (2012). Interferon-γ induces autophagy with growth inhibition and cell death in human hepatocellular carcinoma (HCC) cells through interferon-regulatory factor-1 (IRF-1). Cancer Lett..

[47] Plantureux L., Mège D., Crescence L. (2020). The interaction of platelets with colorectal cancer cells inhibits tumor growth but promotes metastasis. Cancer Res..

[48] Coccia E.M., Del Russo N., Stellacci E. (1999). Activation and repression of the 2-5A synthetase and p21 gene promoters by IRF-1 and IRF-2. Oncogene.

