# Supplementary figures and images for: ITLN1, orchestrated by the IFNγ-IRF1 axis, suppresses hepatocellular carcinoma proliferation via ERK1/2 activation
Source: Transl Oncol. 2025 Nov 11;63:102600. doi: 10.1016/j.tranon.2025.102600 (PMC12651841; doi:10.1016/j.tranon.2025.102600)

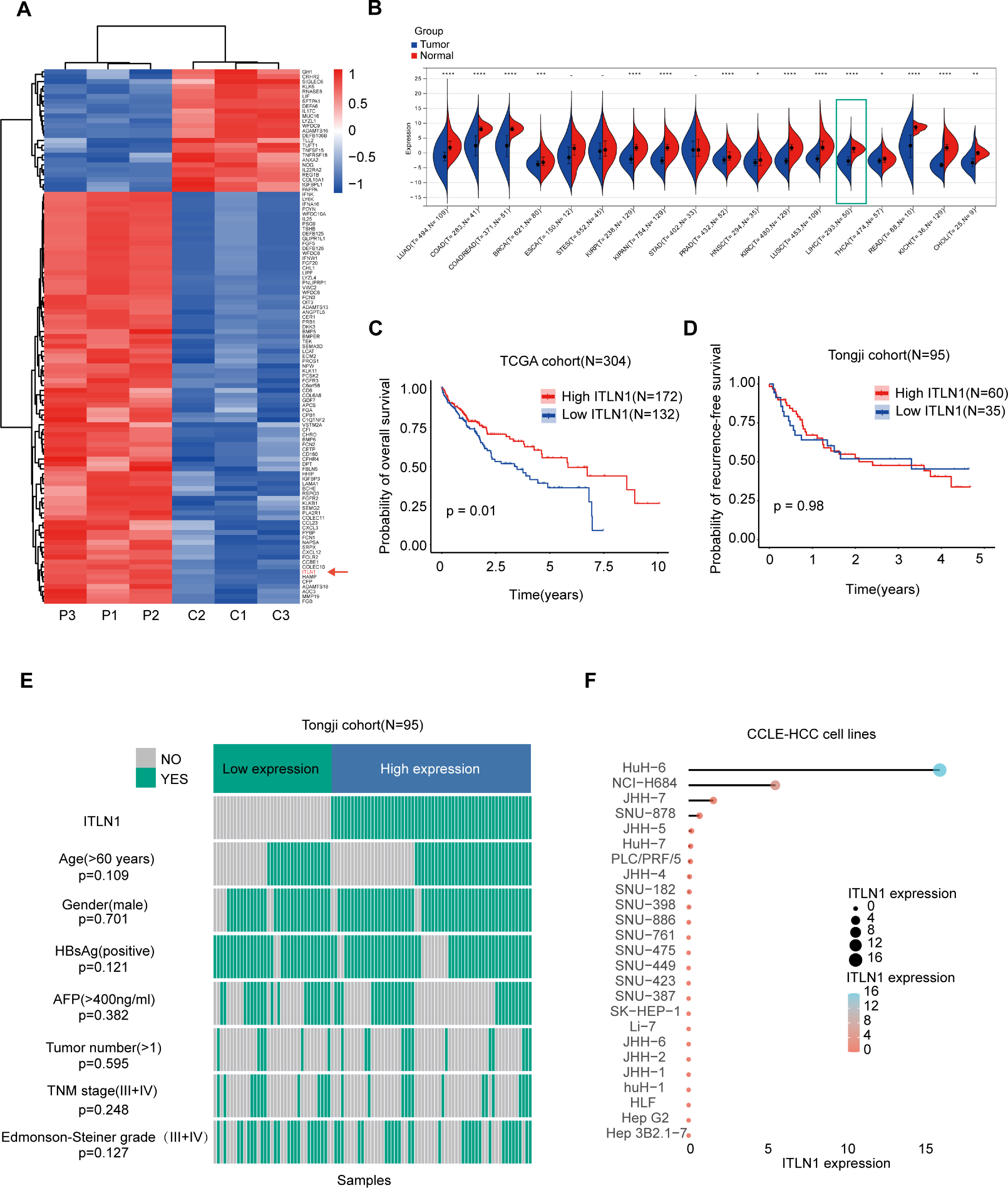

Supplement: Supplementary file 1 [file mmc1.jpg]

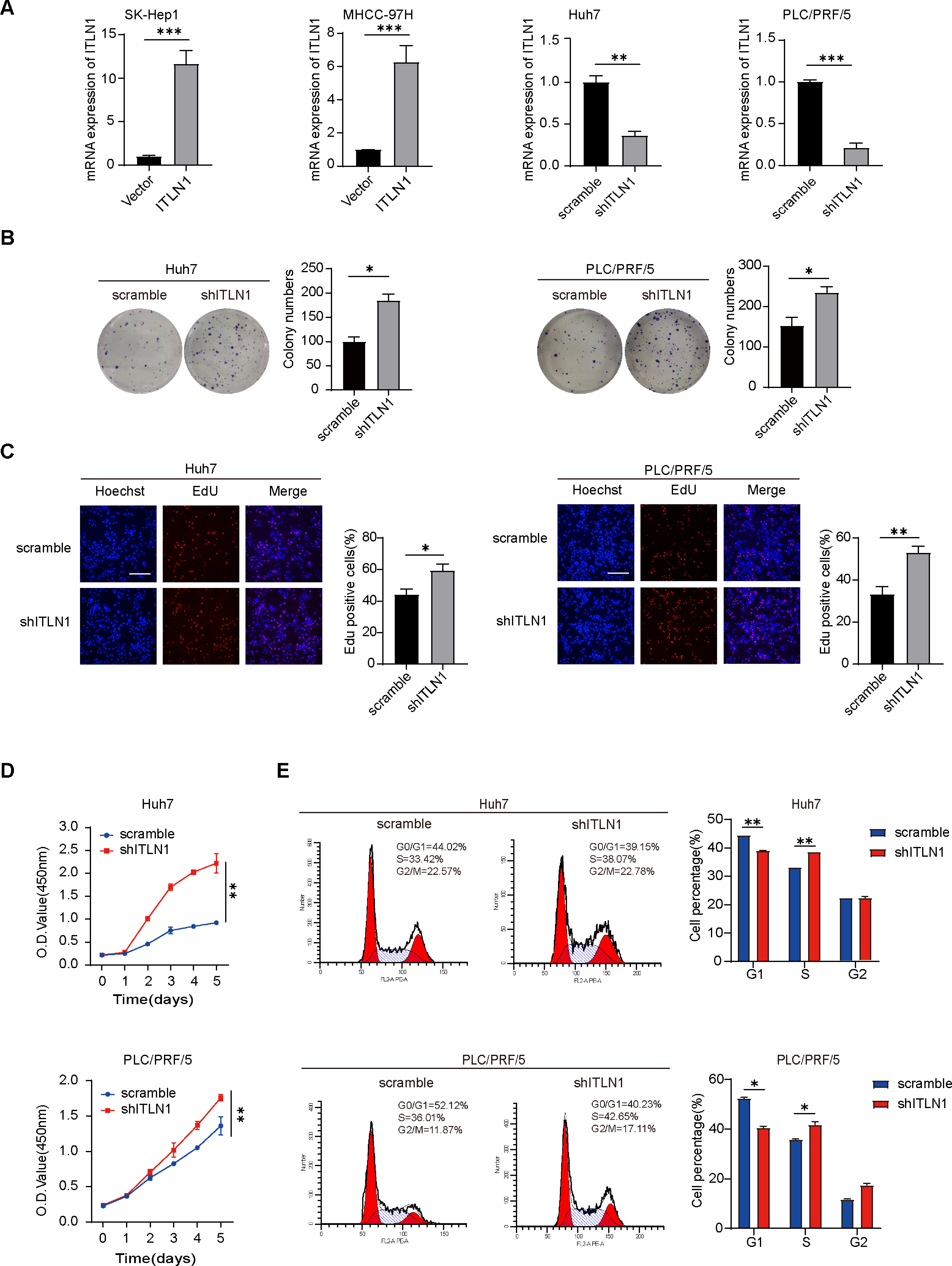

Supplement: Supplementary file 2 [file mmc2.jpg]

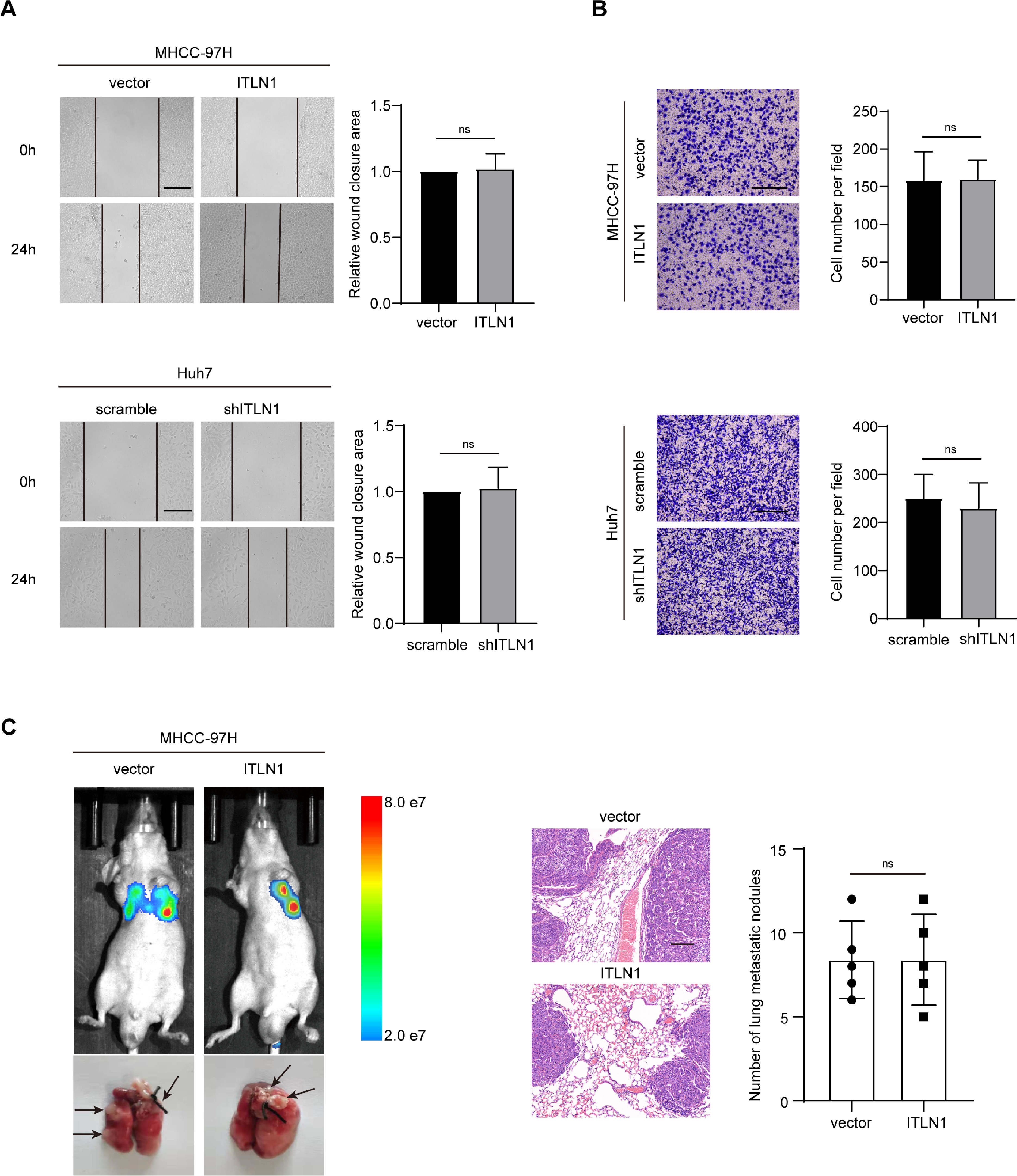

Supplement: Supplementary file 3 [file mmc3.jpg]

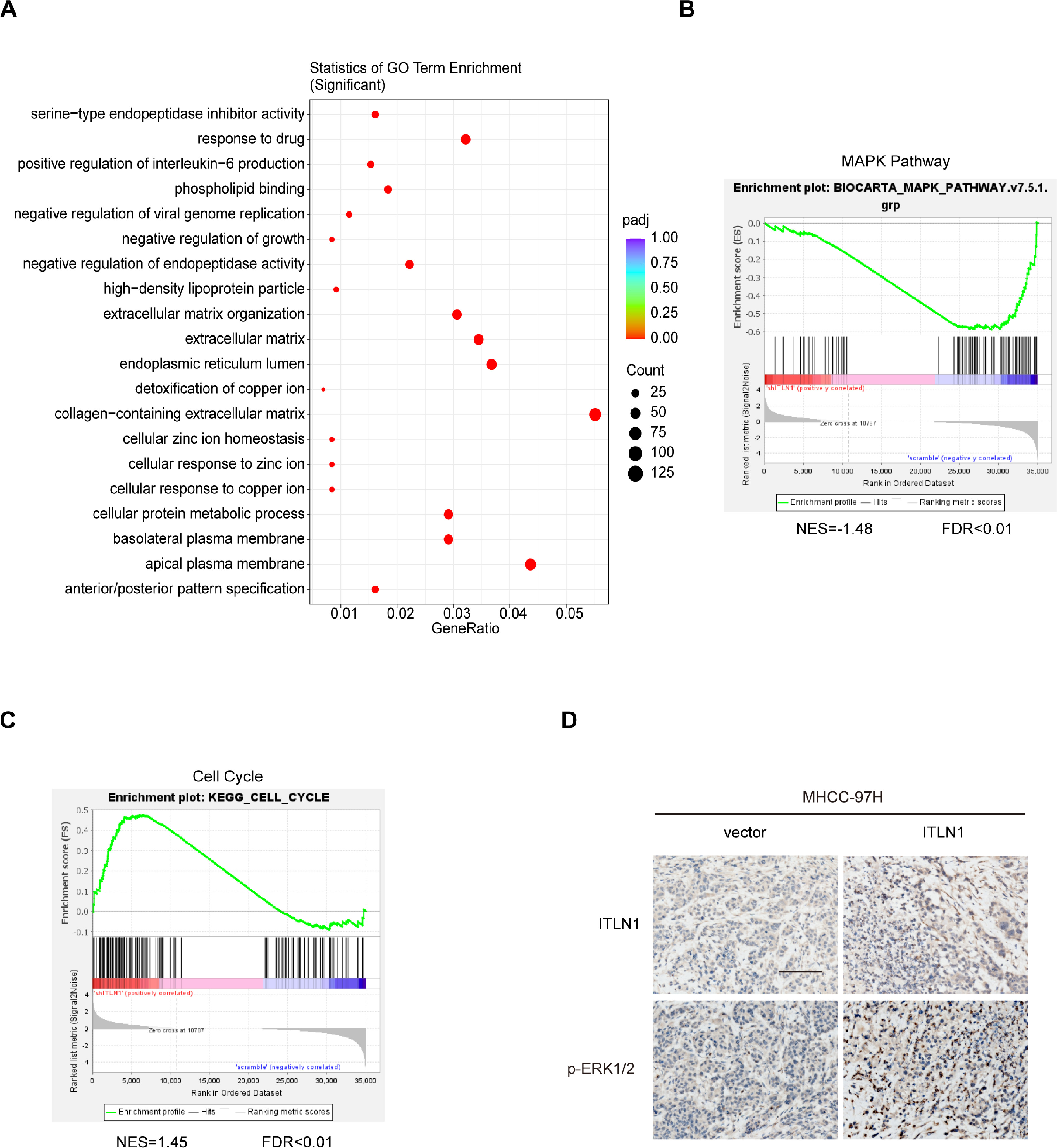

Supplement: Supplementary file 4 [file mmc4.jpg]

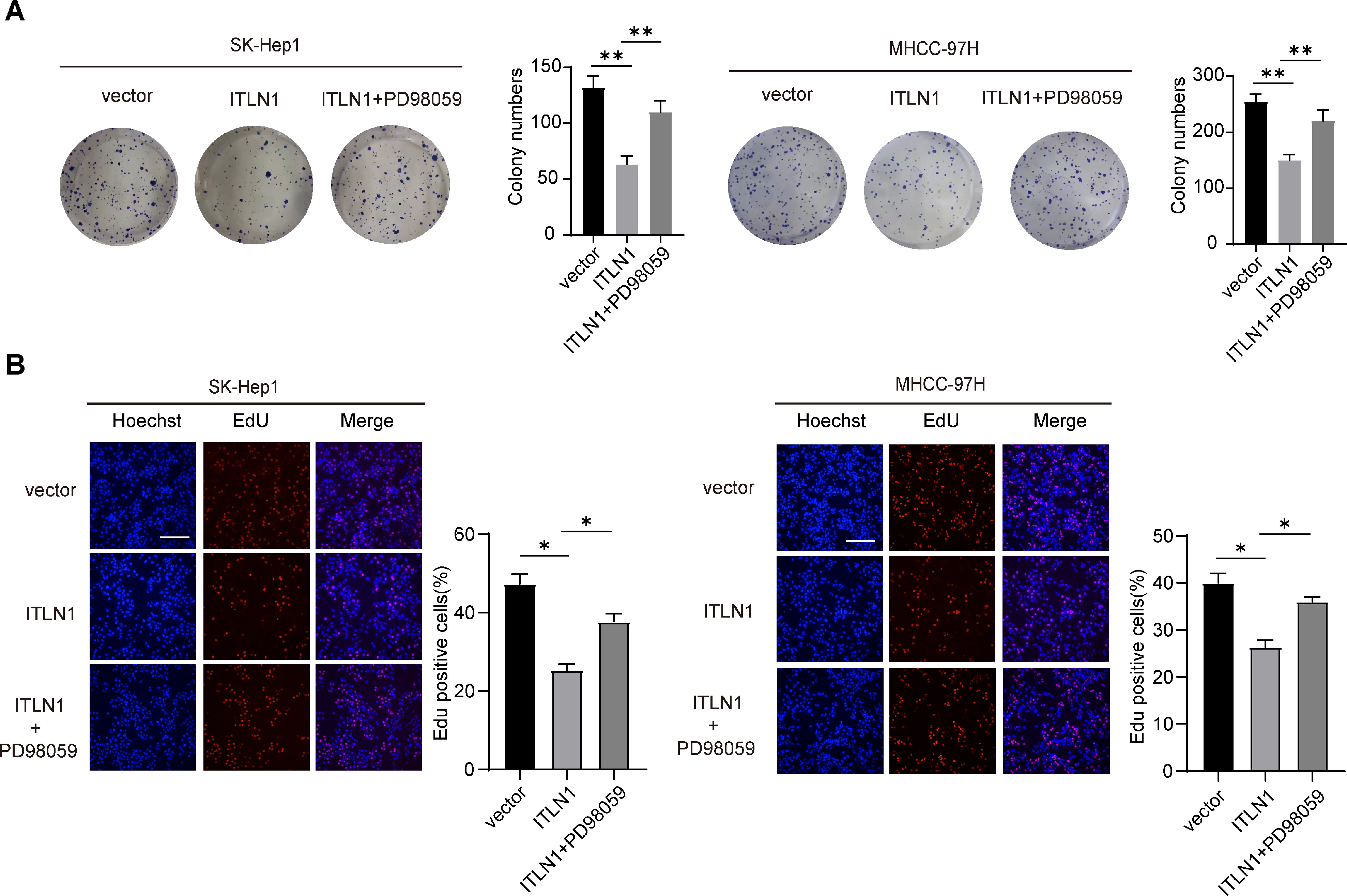

Supplement: Supplementary file 5 [file mmc5.jpg]

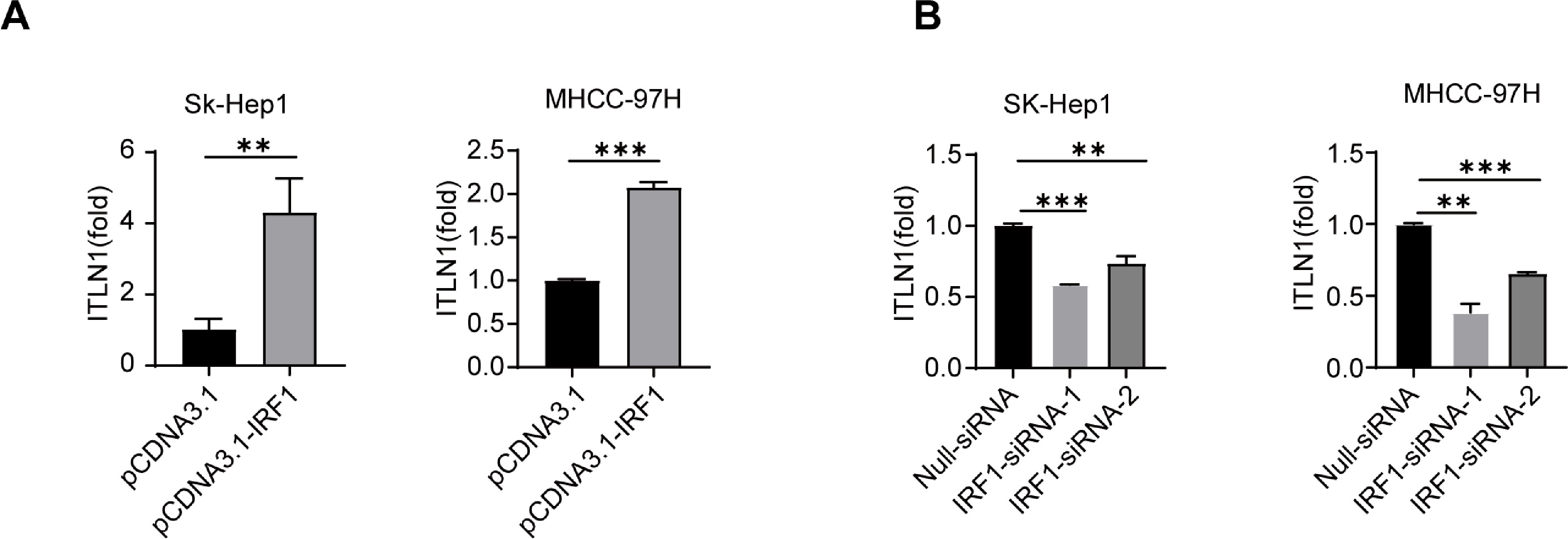

Supplement: Supplementary file 6 [file mmc6.jpg]

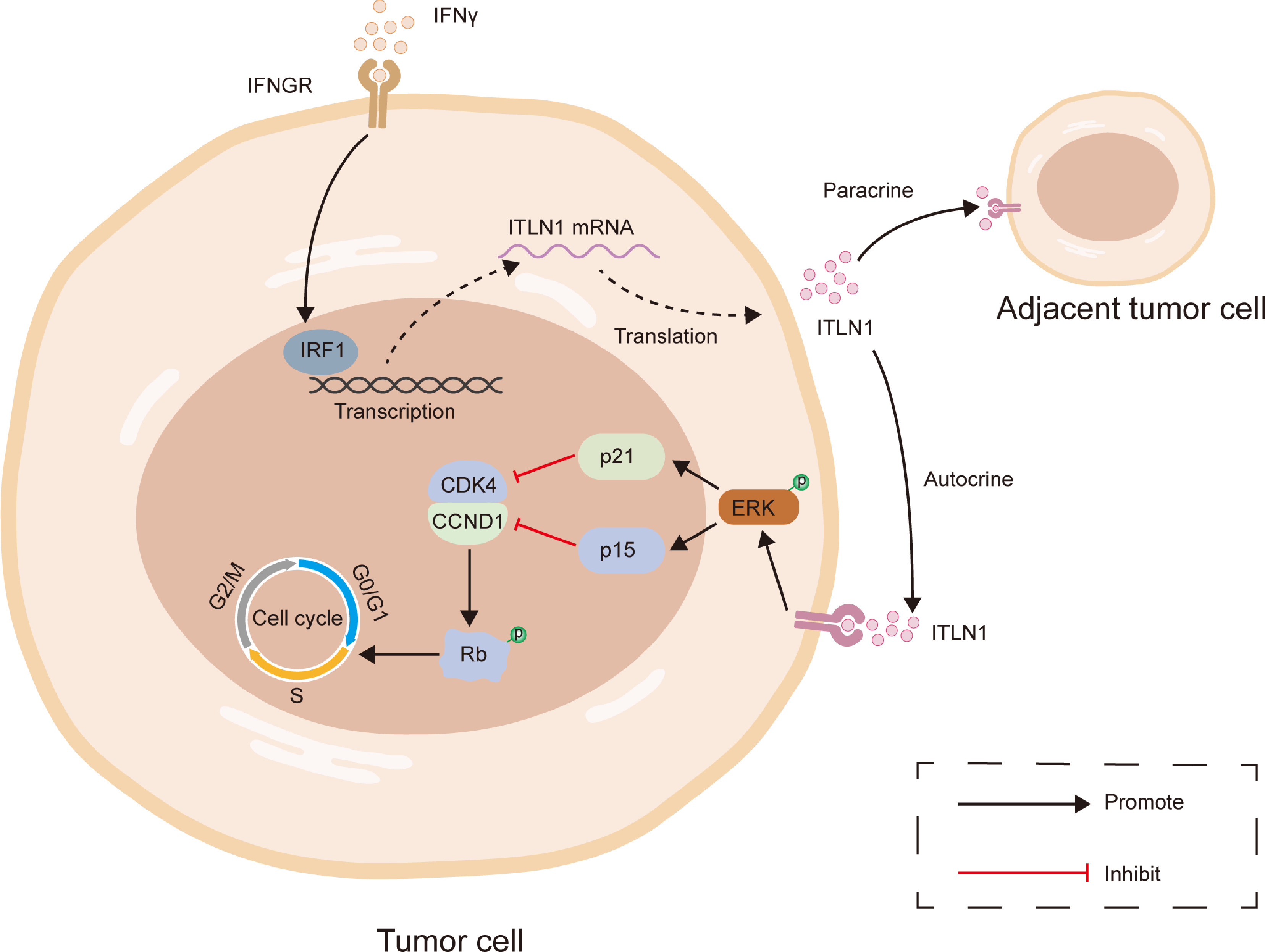

Supplement: Supplementary file 7 [file mmc7.jpg]

Figure 1C

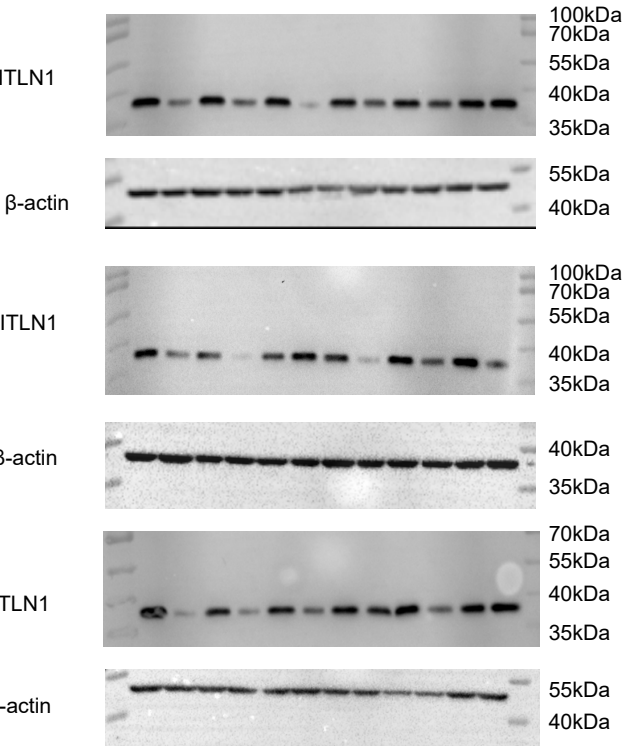

Figure 1H

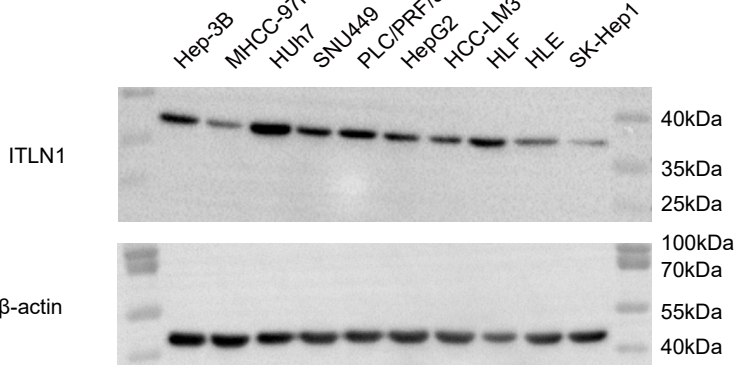

Figure 2A

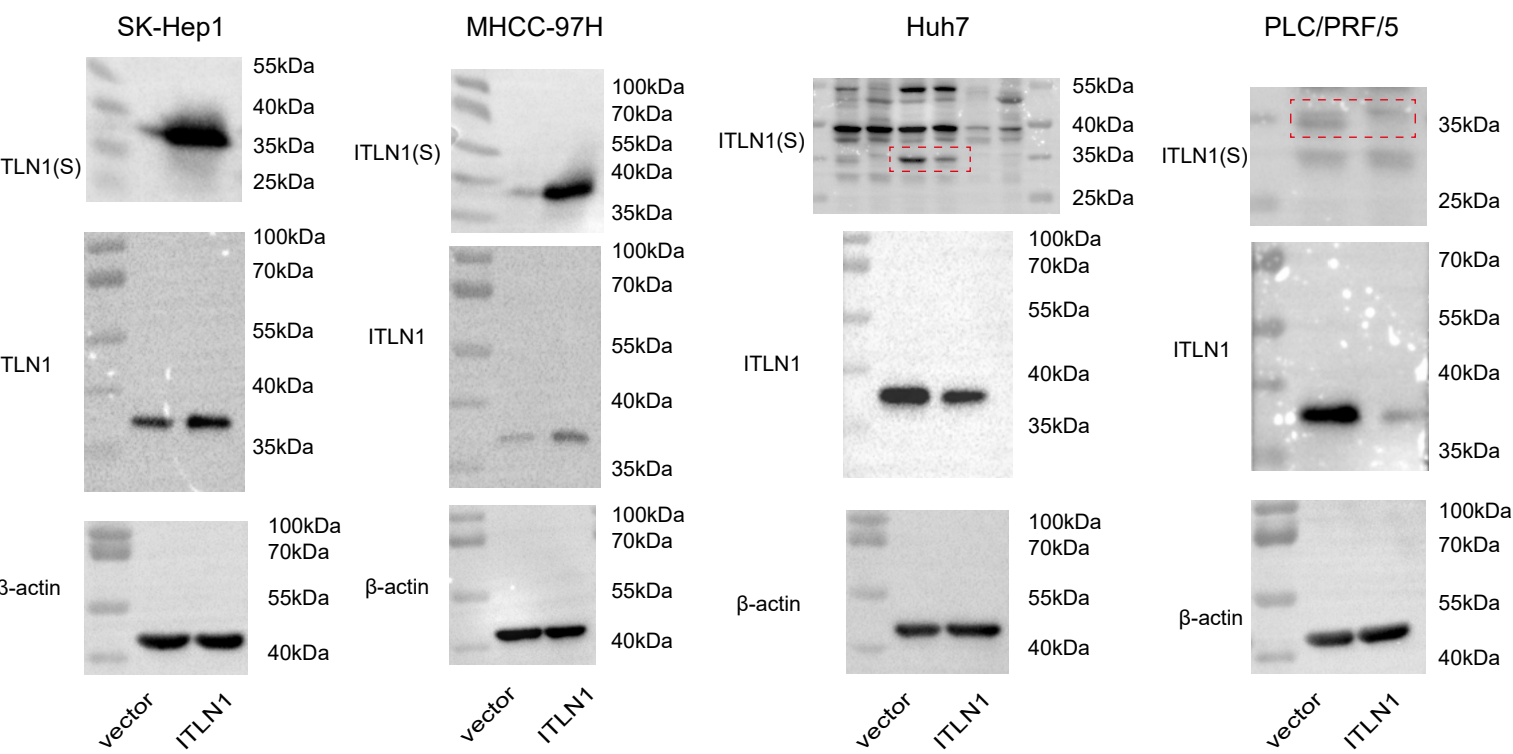

Supplement: Supplementary file 9 [file mmc9.pdf]

Figure 2F

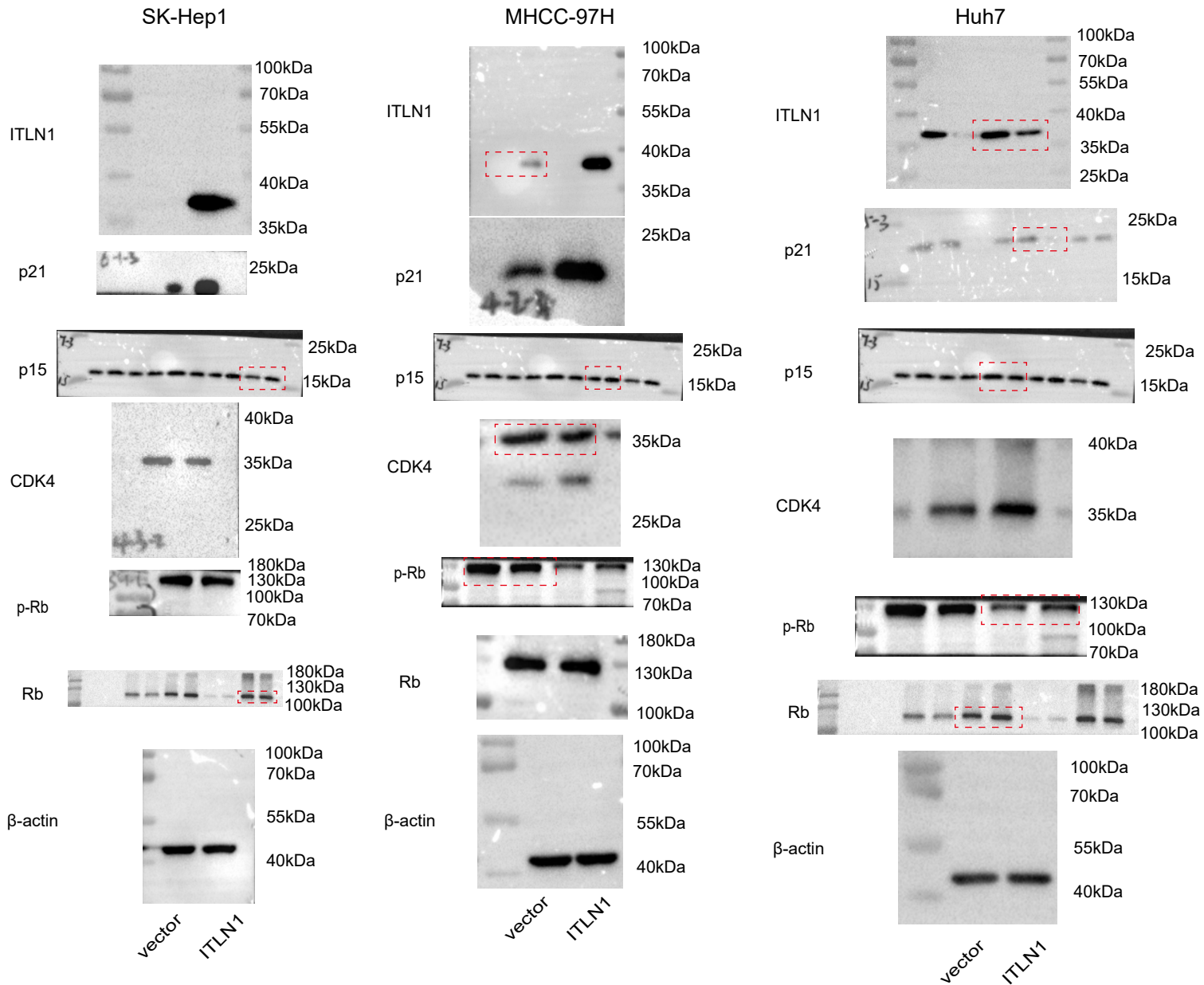

Figure 2F

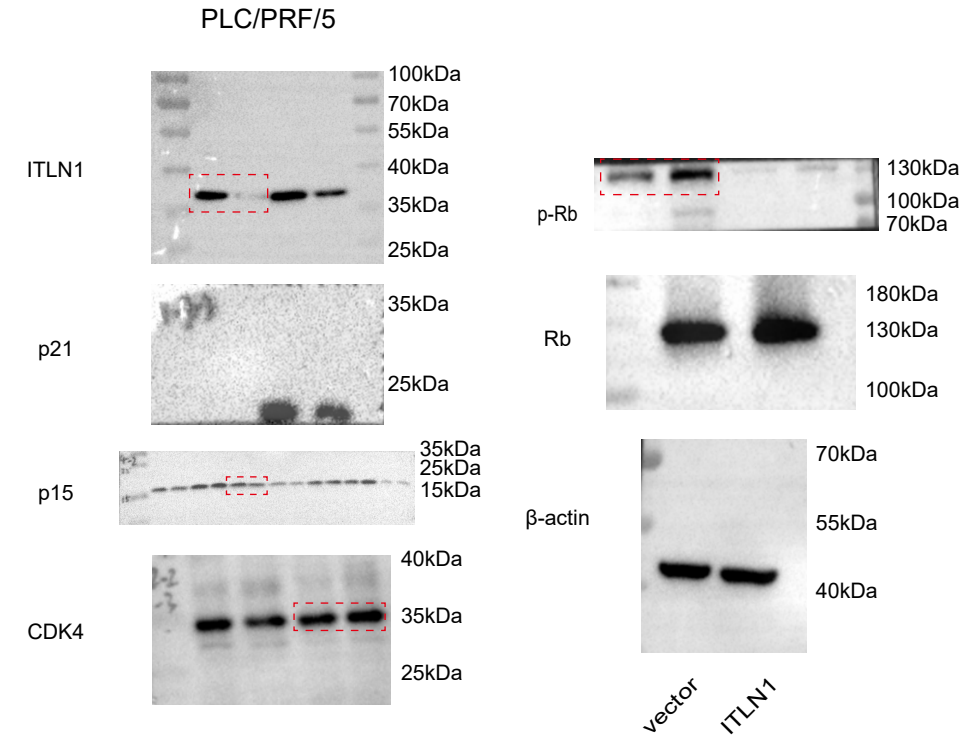

Supplement: Supplementary file 10 [file mmc10.pdf]

Figure 4C

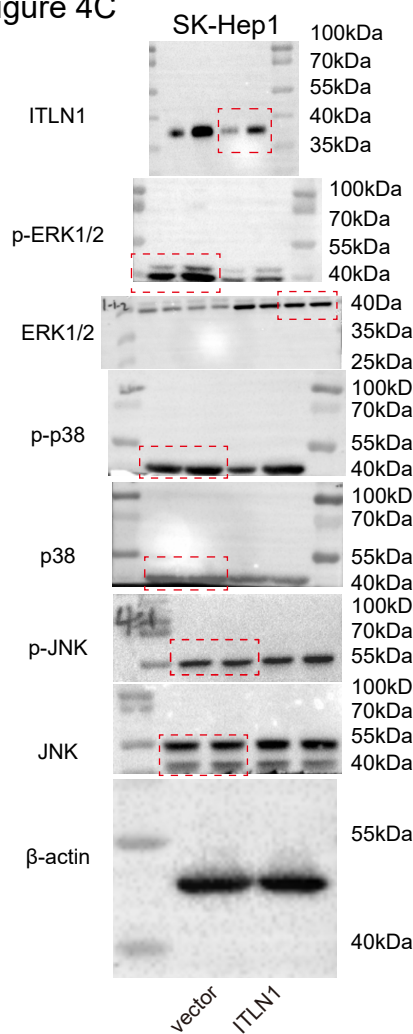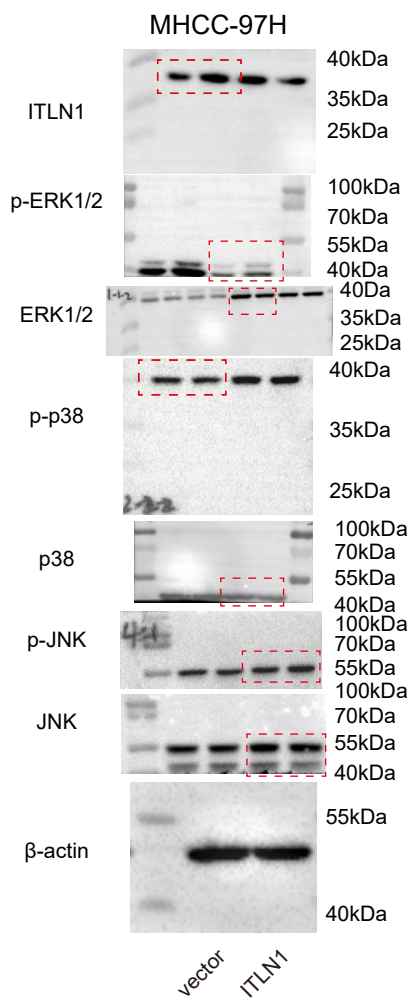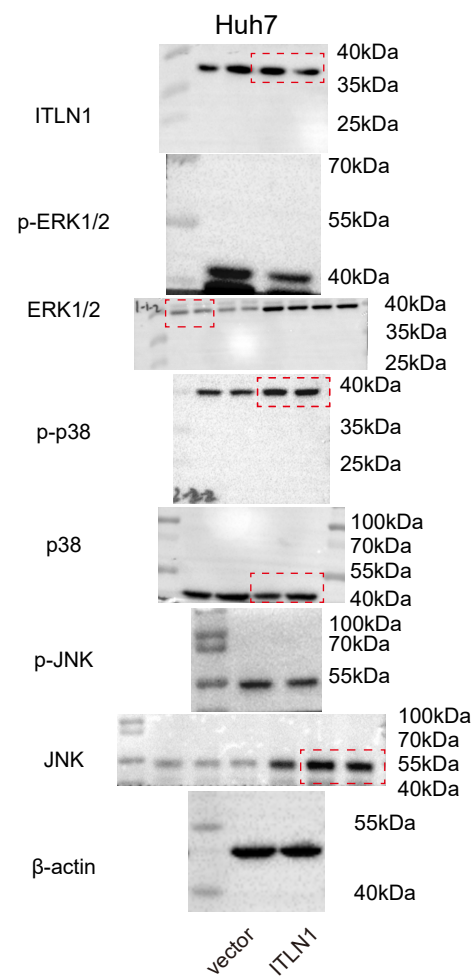

Figure 4C

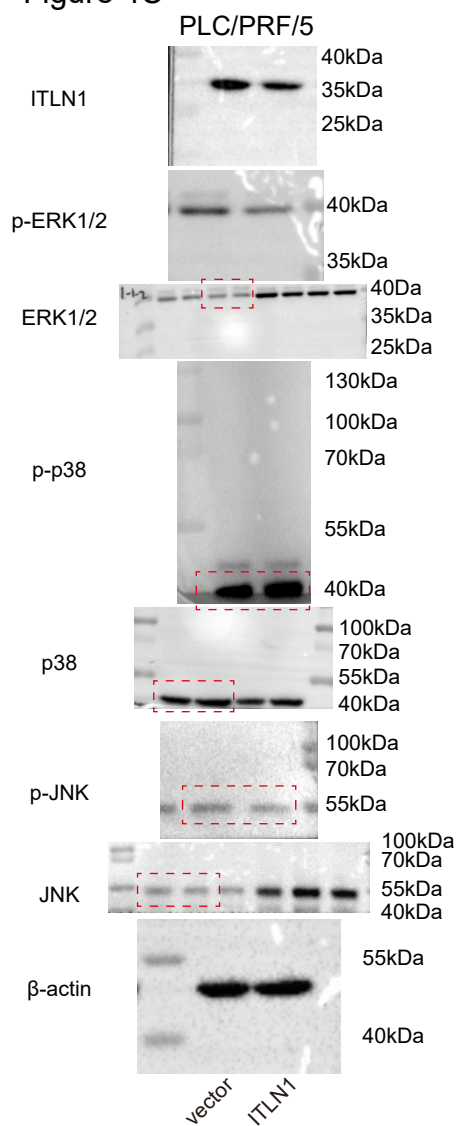

Figure 4D

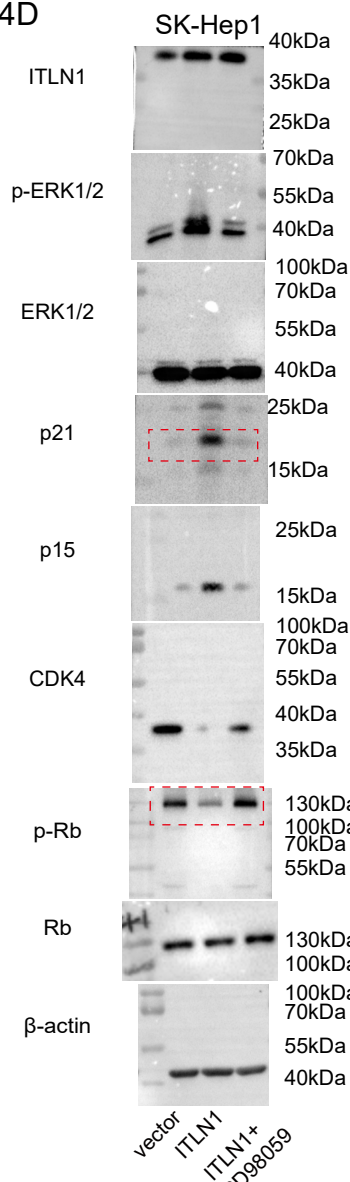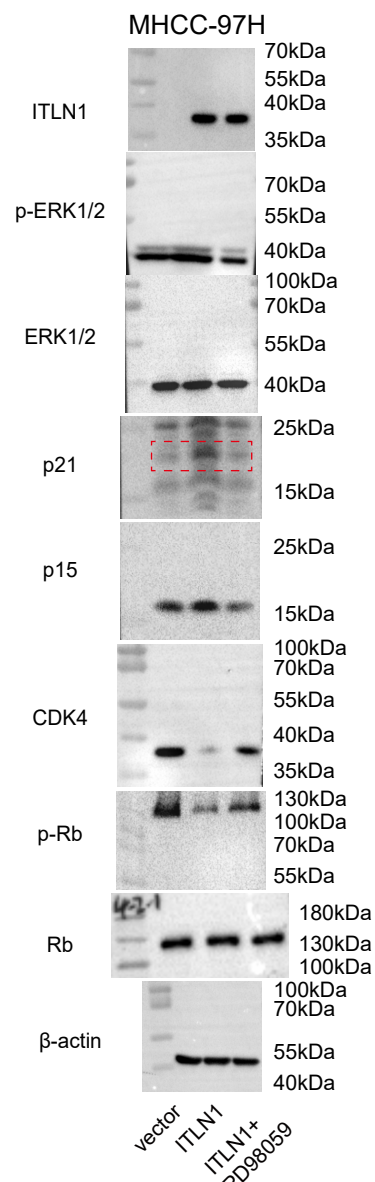

Supplement: Supplementary file 11 [file mmc11.pdf]

Figure 6C

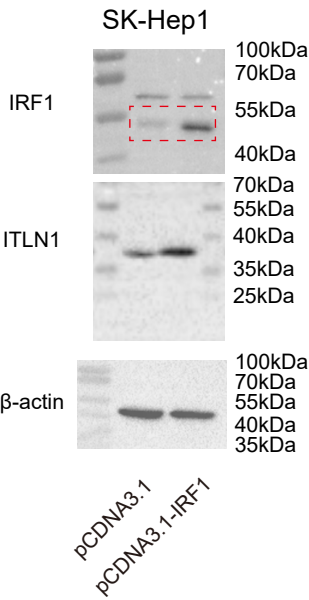

Figure 6D

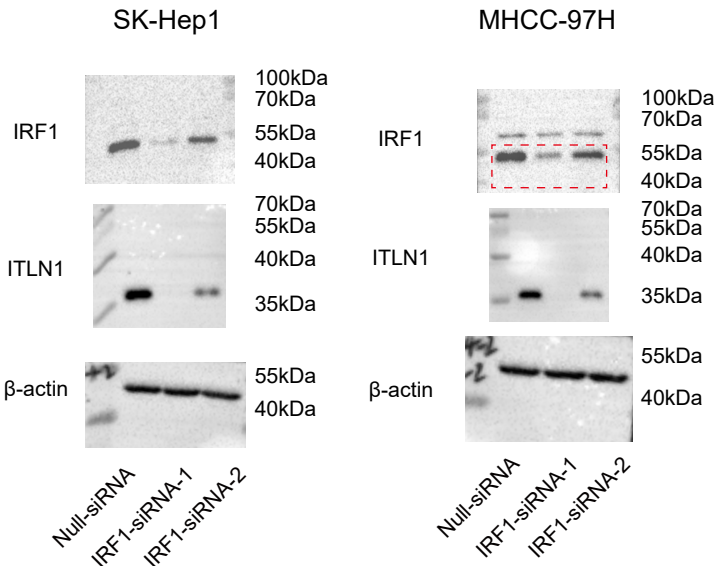

Figure 6E

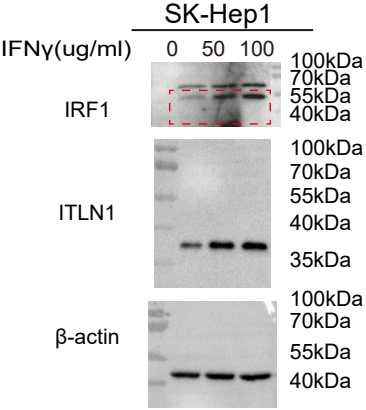

Figure 6F

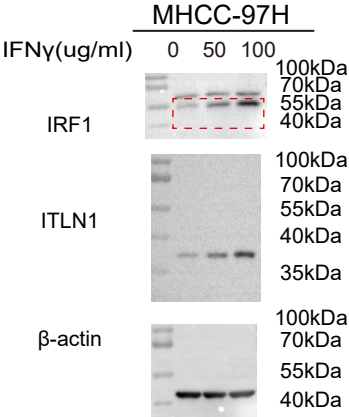

Figure 6G

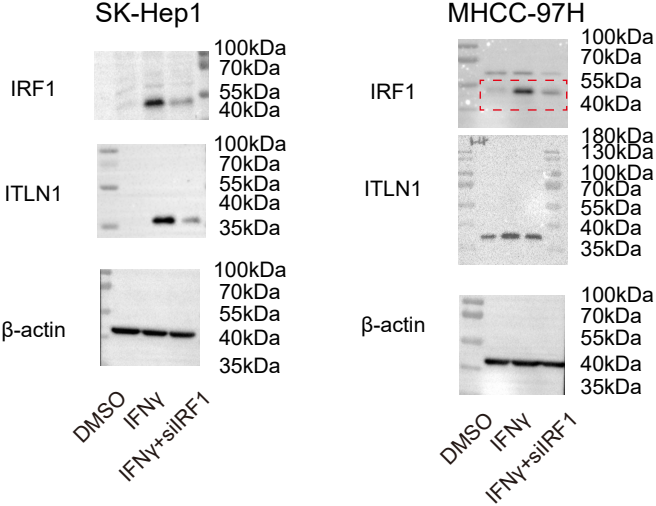

Figure 7A

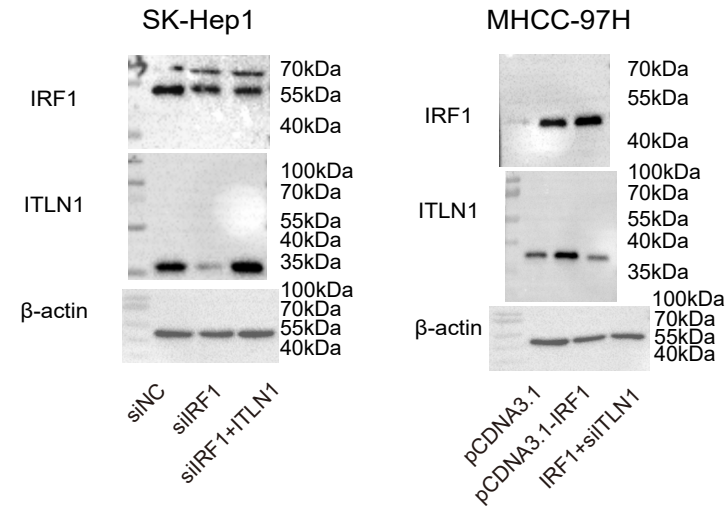

Figure 8A

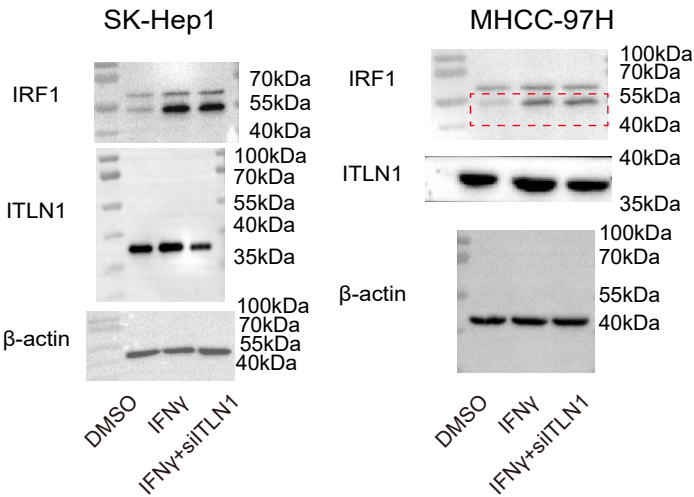

Supplement: Supplementary file 12 — Supporting information [file mmc12.pdf]
