# Supplementary material for: ITLN1, orchestrated by the IFNγ-IRF1 axis, suppresses hepatocellular carcinoma proliferation via ERK1/2 activation
Source: Transl Oncol. 2025 Nov 11;63:102600. doi: 10.1016/j.tranon.2025.102600 (PMC12651841; doi:10.1016/j.tranon.2025.102600)
Supplement: Supplementary file 8 [file mmc8.docx]

**Supplementary tables**

**Table S1 Clinicopathological features of HCC patients in Tongji cohort.**

| Clinicopathological features | Level | Overall |
| --- | --- | --- |
| N (%) |  | 95(100) |
| Age (%) | <=60 years | 41 (43.2) |
|  | >60 years | 54 (56.8) |
| gender (%) | Female | 10 (10.5) |
|  | Male | 85 (89.5) |
| HBsAg status (%) | Negative | 12 (12.6) |
|  | Positive | 83 (87.4) |
| AFP (%) | <=400ng/ml | 49 (51.6) |
|  | >400ng/ml | 46 (48.4) |
| Cirrhosis (%) | Absent | 20 (21.1) |
|  | Present | 75 (78.9) |
| Vascular invasion (%) | Absent | 56 (58.9) |
|  | Present | 39 (41.1) |
| Tumor size (%) | <=5cm | 36 (37.9) |
|  | >5cm | 59 (62.1) |
| Tumor number (%) | Single | 76 (80.0) |
|  | Multiple | 19 (20.0) |
| TNM stage (%) | I+II | 69 (72.6) |
|  | III+IV | 26 (27.4) |
| Edmondson-Steiner grade (%) | I+II | 45 (47.4) |
|  | III+IV | 50 (52.6) |

| **Table S2 Antibodies used in this study.** | | |
| --- | --- | --- |
| Antigens | Manufacturers | Application |
| β-actin | sc-47778, Santa Cruz Biotechnology, Santa Cruz, CA, USA | 1:10,000 for WB |
| anti-rabbit IgG HRP conjugated | Jackson ImmunoResearch Laboratories, Inc. West Grove, PA, USA | 1:5000 for WB |
| anti-mouse IgG HRP conjugated | Jackson ImmunoResearch Laboratories, Inc. West Grove, PA, USA | 1:5000 for WB |
| ITLN1 | Ab252927, Abcam,Cambridge, UK | 1:1000 for WB; 1:50 for IHC |
| p21 | ab109199, Abcam, Cambridge, UK | 1:1000 for WB |
| p15 | ab53034, Abcam, Cambridge, UK | 1:1000 for WB |
| CDK4 | ab108357, Abcam,Cambridge, UK | 1:1000 for WB |
| Rb | #9309,Cell Signaling Technology, Beverly, MA, USA | 1:1000 for WB |
| Phospho-Rb  (Ser807/811) | #8516, Cell Signaling Technology, Beverly, MA, USA | 1:1000 for WB |
| HA-Tag | H6908, Sigma-Aldrich, St. Louis, MO, USA | 1:2000 for WB; 1:50 for ChIP |
| p44/42 MAPK(ERK1/2) | #4695, Cell Signaling Technology,  Beverly, MA, USA | 1:1000 for WB |
| Phospho- p44/42 MAPK(ERK1/2)  (Thr202/Tyr204) | #4370, Cell Signaling Technology,  Beverly, MA, USA | 1:1000 for WB;  1:200 for IHC |
| JNK2 | #9258, Cell Signaling Technology,  Beverly, MA, USA | 1:1000 for WB |
| Phospho-SAPK/JNK (Thr183/Tyr185) | #4668, Cell Signaling Technology,  Beverly, MA, USA | 1:1000 for WB |
| p38 MAPK | #8690, Cell Signaling Technology,  Beverly, MA, USA | 1:1000 for WB |
| Phospho-p38 MAPK (Thr180/Tyr182) | #4511, Cell Signaling Technology,  Beverly, MA, USA | 1:1000 for WB |
| IRF1 | #8478, Cell Signaling Technology, Beverly, MA, USA | 1:1000 for WB; 1:100 for IHC; 1:50 for ChIP |
| IFNγ | #DF6045, Affinity Biosciences LTD,  Melbourne, Au | 1:50 for IHC |
| IHC, immunohistochemistry; WB, western blot; ChIP, chromatin immunoprecipitation. | | |

**Table S3 shRNA and siRNA sequences.**

| Identifier | Sequence |
| --- | --- |
| scramble-forward | 5'-CCGGCCTAAGGTTAAGTCGCCCTCGCTCGAG  CGAGGGCGACTTAACCTTAGGTTTTT-3' |
| scramble-reverse | 5'-AATTAAAAACCTAAGGTTAAGTCGCCCTCGCTCG  AGCGAGGGCGACTTAACCTTAGG-3' |
| shITLN1-forward | 5'-CCGGGCATCTTATTACTCACCCTATCTCGAGATA  GGGTGAGTAATAAGATGCTTTTT-3' |
| shITLN1-reverse | 5'-AATTAAAAAGCATCTTATTACTCACCCTATCTCGA  GATAGGGTGAGTAATAAGATGC-3' |
| ITLN1-siRNA | 5'-GCATCTTATTACTCACCCTAT-3' |
| IRF1-siRNA-1 | 5'-GCGTGTCTTCACAGATCTGAA-3' |
| IRF1-siRNA-2 | 5'-GCAGATTAATTCCAACCAAAT-3' |

**Table S4 Sequences of gene-specific primers used for qRT-PCR and ChIP.**

| Primer set | Primers | Sequence | Product size(bp) | Application |
| --- | --- | --- | --- | --- |
| ITLN1 | Forward | 5'-AGTGTTGGACTGACAACGGC-3' | 186 | qPCR |
|  | Reverse | 5'-TACATCCGGTGACCCTCATTC-3' |  |  |
| β-actin | Forward | 5'-CATGTACGTTGCTATCCAGGC-3' | 250 | qPCR |
|  | Reverse | 5'-CTCCTTAATGTCACGCACGAT-3' |  |  |
| IRF1 | Forward | 5'-ATGCCCATCACTCGGATGC-3' | 204 | qPCR |
|  | Reverse | 5'-CCCTGCTTTGTATCGGCCTG-3' |  |  |
| IFNγ | Forward | 5'-TCGGTAACTGACTTGAATGTCCA-3' | 93 | qPCR |
|  | Reverse | 5'-TCGCTTCCCTGTTTTAGCTGC-3' |  |  |
| p21 | Forward | 5'-CGATGGAACTTCGACTTTGTCA-3' | 220 | qPCR |
|  | Reverse | 5'-GCACAAGGGTACAAGACAGTG-3' |  |  |
| p15 | Forward | 5'-CGCCCACAACGACTTTATTT-3' | 184 | qPCR |
|  | Reverse | 5'-CGAGGGCCAGATAAGACAAA-3' |  |  |
| site 1 | Forward | 5'-GATCTTGGGCCCTTGGTGTT-3' | 70 | ChIP |
|  | Reverse | 5'- GAGGTCTCTAAGTTTACAGTTCACA-3' |  |  |
| site 2 | Forward | 5'-TCCAGATACACAGAGCAAATAGC-3' | 152 | ChIP |
|  | Reverse | 5'-CCTATTTGTTGGTTCAAAGTACTGA-3' |  |  |
| site 3 | Forward | 5'-AAGTAGTGAGTCTTTGTGTGAAACT-3' | 124 | ChIP |
|  | Reverse | 5'-TTGGTAAAAAGGAATGGAGGGG-3' |  |  |
| site 4 | Forward | 5'-AAGGCACAAAAACCCAAAACCAA-3' | 73 | ChIP |
|  | Reverse | 5'-TGGACCTGTGTCAAGTAGGAAG-3' |  |  |

ITLN1, intelectin 1; β-actin, actin beta ; IRF1, interferon regulatory factor 1; IFNγ, interferon-gamma; p21, Cyclin Dependent Kinase Inhibitor 1A; p15, Cyclin- Dependent Kinase Inhibitor 2B; site 1, ITLN1 promoter binding stie 1; site 2, ITLN1 promoter binding stie 2; site 3, ITLN1 promoter binding stie 3; site 4, ITLN1 promoter binding stie 4.

**Table S5 One hundred and nine differentially expressed secretory proteins between HCC tumor and adjacent non-tumor tissues.**

| Genbank Accession | Gene Symbol | Fold change |
| --- | --- | --- |
| NM_001002857 | ANXA2 | 10.34902 |
| NM_001040704 | DEFB106B | 8.270454 |
| NM_006507 | REG1B | 7.710201 |
| NM_147198 | WFDC9 | 7.335638 |
| NM_001855 | COL15A1 | 6.722294 |
| NM_005118 | TNFSF15 | 4.785714 |
| NM_032517 | LYZL1 | 4.271633 |
| NM_148901 | TNFRSF18 | 3.822662 |
| NM_024690 | MUC16 | 3.641073 |
| NM_005450 | NOG | 3.211748 |
| NM_052962 | IL22RA2 | 3.176521 |
| NM_139056 | ADAMTS16 | 3.097435 |
| NM_002309 | LIF | 2.882056 |
| NM_013278 | IL17C | 2.820596 |
| NM_020127 | TUFT1 | 2.575144 |
| NM_001883 | CRHR2 | 2.312852 |
| NM_138331 | RNASE8 | 2.282795 |
| NM_001926 | DEFA6 | 2.19604 |
| NM_002581 | PAPPA | 2.194885 |
| NM_000515 | GH1 | 2.175472 |
| NM_012465 | TLL2 | 2.16545 |
| NM_001012964 | KLK6 | 2.148155 |
| NM_005411 | SFTPA1 | 2.129836 |
| NM_001007563 | IGFBPL1 | 2.040234 |
| NM_001245 | SIGLEC6 | 2.027515 |
| NM_080827 | WFDC6 | 0.491256 |
| NM_144634 | LYZL4 | 0.482596 |
| NM_004190 | LIPF | 0.480288 |
| NM_198570 | VWC2 | 0.470231 |
| NM_003008 | SEMG2 | 0.469846 |
| NM_002594 | PCSK2 | 0.469116 |
| NM_024411 | PDYN | 0.455187 |
| NM_005454 | CER1 | 0.452995 |
| U32331 | DKK3 | 0.447006 |
| NM_005039 | PRB1 | 0.445055 |
| NM_030931 | DEFB126 | 0.444938 |
| NM_001010905 | C6orf58 | 0.443706 |
| NM_130896 | WFDC8 | 0.440823 |
| NM_006229 | PNLIPRP1 | 0.435563 |
| NM_002173 | IFNA16 | 0.430525 |
| NM_006614 | CHL1 | 0.424734 |
| NM_017527 | LY6K | 0.423803 |
| NM_019851 | FGF20 | 0.421435 |
| NM_002177 | IFNW1 | 0.413879 |
| NM_003741 | CHRD | 0.406652 |
| NM_020124 | IFNK | 0.401132 |
| XM_006715663 | VSTM2A | 0.392523 |
| NM_153325 | DEFB125 | 0.39109 |
| NM_002784 | PSG9 | 0.388492 |
| NM_004464 | FGF5 | 0.387098 |
| NM_022789 | IL25 | 0.382233 |
| NM_005559 | LAMA1 | 0.382078 |
| NM_080753 | WFDC10A | 0.378061 |
| S51112 | TSHB | 0.362044 |
| NM_003734 | AOC3 | 0.346714 |
| NM_152779 | GLIPR1L1 | 0.335667 |
| NM_000803 | FOLR2 | 0.329733 |
| NM_000313 | PROS1 | 0.319543 |
| NM_006725 | CD6 | 0.319043 |
| NM_144947 | KLK11 | 0.317781 |
| NM_005141 | FGB | 0.262968 |
| NM_031908 | C1QTNF2 | 0.255918 |
| NM_001639 | APCS | 0.250517 |
| NM_004851 | NAPSA | 0.24423 |
| NM_199355 | ADAMTS18 | 0.23606 |
| NM_001718 | BMP6 | 0.228521 |
| NM_000142 | FGFR3 | 0.219611 |
| M87772 | FGFR2 | 0.215087 |
| NM_002090 | CXCL3 | 0.209942 |
| NM_002429 | MMP19 | 0.196048 |
| NM_152754 | SEMA3D | 0.188427 |
| NM_001871 | CPB1 | 0.182666 |
| NM_000204 | CFI | 0.173886 |
| NM_000508 | FGA | 0.168947 |
| NM_002003 | FCN1 | 0.15395 |
| NM_006329 | FBLN5 | 0.153933 |
| NM_002704 | PPBP | 0.150139 |
| NM_001013398 | IGFBP3 | 0.138891 |
| NM_000459 | TEK | 0.133262 |
| NM_182828 | GDF7 | 0.130934 |
| NM_000892 | KLKB1 | 0.113945 |
| NM_032784 | RSPO3 | 0.104315 |
| NM_021073 | BMP5 | 0.097578 |
| NM_199235 | COLEC11 | 0.096631 |
| BC036806 | ECM2 | 0.096148 |
| NM_031917 | ANGPTL6 | 0.095156 |
| NM_001937 | DPT | 0.0947 |
| NM_007366 | PLA2R1 | 0.087939 |
| NM_139025 | ADAMTS13 | 0.072845 |
| NM_006684 | CFHR4 | 0.06368 |
| NM_007053 | CD160 | 0.062062 |
| NM_000055 | BCHE | 0.046042 |
| NM_001102608 | COL6A6 | 0.04584 |
| NM_000229 | LCAT | 0.043525 |
| NM_001099456 | NPW | 0.043293 |
| NM_199168 | CXCL12 | 0.035528 |
| NM_006307 | SRPX | 0.035114 |
| NM_005064 | CCL23 | 0.026877 |
| NM_133468 | BMPER | 0.025536 |
| NM_000078 | CETP | 0.023249 |
| NM_006438 | COLEC10 | 0.022908 |
| NM_002621 | CFP | 0.018606 |
| NM_133459 | CCBE1 | 0.010825 |
| NM_003665 | FCN3 | 0.008237 |
| AK124396 | HHIP | 0.005121 |
| NM_017625 | ITLN1 | 0.004538 |
| NM_021175 | HAMP | 0.003692 |
| NM_004108 | FCN2 | 0.002416 |
| NM_152635 | OIT3 | 0.001376 |

**Table S6** **Possible transcription factor binding sites within the 2.1 kilobases promoter region of ITLN1 were predicted using the JASPAR database.**

| Ensembl | Factor name |
| --- | --- |
| ENSG00000124782 | RREB1 |
| ENSG00000125347 | IRF1 |
| ENSG00000147862 | NFIB |
| ENSG00000197714 | ZNF460 |
| ENSG00000176293 | ZNF135 |
| ENSG00000141905 | NFIC |
| ENSG00000008441 | NFIX |
| ENSG00000275410 | HNF1B |
| ENSG00000109132 | PHOX2B |
| ENSG00000066336 | SPI1 |
| ENSG00000168267 | PTF1A |
| ENSG00000135100 | HNF1A |
| ENSG00000163435 | ELF3 |
| ENSG00000126746 | ZNF384 |
| ENSG00000196646 | ZNF136 |
| ENSG00000078900 | TP73 |
| ENSG00000165462 | PHOX2A |
| ENSG00000175325 | PROP1 |
| ENSG00000164920 | OSR2 |
| ENSG00000166888 | STAT6 |
| ENSG00000187140 | FOXD3 |
| ENSG00000185669 | SNAI3 |
